# Optimizing Green Extraction Methods for Maximizing the Biological Potential of Dandelion, Milk Thistle, and Chamomile Seed Extracts

**DOI:** 10.3390/foods13233907

**Published:** 2024-12-03

**Authors:** Stoja Milovanovic, Katarzyna Tyśkiewicz, Marcin Konkol, Agnieszka Grzegorczyk, Kinga Salwa, Łukasz Świątek

**Affiliations:** 1 Faculty of Technology and Metallurgy, University of Belgrade, Karnegijeva 4, 11120 Belgrade, Serbia; 2 Łukasiewicz Research Network—New Chemical Syntheses Institute, Al. Tysiąclecia Państwa Polskiego 13a, 24-110 Puławy, Poland; katarzyna.tyskiewicz@ins.lukasiewicz.gov.pl (K.T.); marcin.konkol@ins.lukasiewicz.gov.pl (M.K.); 3 Chair and Department of Pharmaceutical Microbiology, Medical University of Lublin, Chodźki 1, 20-093 Lublin, Poland; agnieszka.grzegorczyk@umlub.pl; 4 Department of Virology with Viral Diagnostics Laboratory, Medical University of Lublin, Chodźki 1, 20-093 Lublin, Poland; kinga.salwa@umlub.pl (K.S.); lukasz.swiatek@umlub.pl (Ł.Ś.)

**Keywords:** supercritical carbon dioxide extraction, edible oils isolation, antimicrobial activity, antioxidant activity, cytotoxicity, biological activity, phytochemical isolation, natural food resources

## Abstract

This study investigates the underutilized potential of agri-crops from the *Asteraceae* family by employing sustainable and green technologies (supercritical fluid, ultrasound, and Soxhlet extractions) to enhance the recovery of bioactive compounds. A total of 21 extracts from native and waste seeds of dandelion, milk thistle, and chamomile were systematically compared utilizing a combination of solvents (supercritical CO_2_ and absolute or aqueous ethanol). Supercritical CO_2_ extraction yielded up to 281 mg/g of oils from native seeds, while conventional techniques with ethanol recovered an additional 142 mg/g of extracts from waste seeds. Notably, waste seed extracts exhibited superior biological activity, including potent antioxidant properties (IC_50_ values as low as 0.3 mg/mL in the DPPH assay) and broad-spectrum antimicrobial activity against 32 microbial strains, including methicillin-resistant *Staphylococcus aureus*, Gram-negative bacteria, and yeast strains. Phenolic compounds were abundant, with up to 2126 mg GAE/g, alongside 25.9 mg QE/g flavonoids, and 805.5 mg/kg chlorophyll A. A selective anticancer activity of waste milk thistle extracts was observed, with a selectivity index of 1.9 to 2.7. The oils recovered from native seeds demonstrated lower bioactivity and are well-suited for applications in food. The potent bioactivity of the smaller quantities of waste seed extracts positions them as valuable candidates for pharmaceutical use.

## 1. Introduction

Dandelion, milk thistle, and chamomile, all members of the *Asteraceae* family, are commercially cultivated agri-crops that are Generally Recognized as Safe (GRAS) by the U.S. Food and Drug Administration (FDA) for food and supplement use [1,2,3]. Historically, the flowers of these plants have been used in functional teas—dandelion for its diuretic properties [4], milk thistle for liver health [5], and chamomile for digestive ailments [6]. The beneficial health effects of these plants are attributed to various bioactive compounds, including phenolics, sterols, polysaccharides, fatty acids, minerals, and vitamins [1,4,5,6]. Extracts from these plants have demonstrated antioxidant, antimicrobial, and anti-inflammatory properties, as well as anticancer potential [1,2,4,5,6]. Furthermore, the dietary intake of their extracts has been linked to the prevention of diseases such as obesity, diabetes, cardiovascular disease, and liver disorders [2,4,7].

While flower extracts have received considerable attention, the seeds of dandelion, milk thistle, and chamomile, are also a rich source of bioactive compounds, present significant opportunities for pharmaceutical, cosmetic, and food industries [8]. Seed extracts can be obtained via several methods, including traditional solvent extraction using Soxhlet apparatus or more sustainable approaches like ultrasound-assisted extraction (UAE) and supercritical fluid extraction (SFE). Soxhlet extraction (SXE), though effective, often requires high temperatures and large quantities of solvents, posing environmental challenges due to solvent waste and the potential degradation of thermolabile compounds [8,9,10]. UAE, on the other hand, offers faster extractions and lower energy consumption, enhancing recovery efficiency [5,8,11]. However, both methods still rely on post-extraction steps like filtration and solvent evaporation. SFE with supercritical carbon dioxide (sc-CO_2_) represents a greener alternative, especially for extracting oils from seeds. This method operates at moderate temperatures, eliminating the need for organic solvents and producing solvent-free extracts suitable for the food and pharmaceutical industries [8,10,12,13]. However, because sc-CO_2_ is non-polar, polar compounds remain in the residual plant material, necessitating further extraction with polar solvents [14,15,16].

Given the increasing demand for bioactive products derived from plant resources, the global market for herbal extract products was valued at USD 27.9 billion in 2022 and is projected to grow to USD 85.1 billion by 2032 [17]. Specifically, the market for milk thistle products is expected to reach USD 234.2 million by 2030 [18], while the chamomile and dandelion extracts markets are projected to grow significantly as well [19,20]. Despite the rising commercial use of extracts from milk thistle seeds, their production still predominantly relies on organic solvent-based methods, whereas dandelion and chamomile seed extracts are less common, especially in European markets. This study aims to meet these market trends and sustainability demands by investigating the extraction of bioactive compounds from both native and waste seeds of dandelion, milk thistle, and chamomile. Previous research has optimized SFE processes for native seeds, focusing on temperature and pressure variations [21,22,23], leaving the potential of waste seed valorization largely unexplored. The current study extends this work by applying green extraction techniques (SFE, SXE, and USE) with environmentally friendly solvents (absolute ethanol, aqueous ethanol, and sc-CO_2_). To our knowledge, reports on the extraction of bioactive compounds from the seeds of mentioned plants, particularly using sustainable solvents and techniques, remain scarce. Furthermore, the valorization of waste seed materials generated during SFE represents an untapped opportunity in both the food and pharmaceutical industries. Waste valorization is a particularly impactful innovation because it aligns with principles of sustainability and the circular economy, turning byproducts into high-value resources. This approach minimizes resource wastage and supports global efforts to reduce environmental footprints. Furthermore, this work aligns directly with several United Nations Sustainable Development Goals (SDGs) specifically: SDG 3 (Good Health and Well-being), SDG 9 (Industry, Innovation, and Infrastructure), and SDG 12 (Responsible Consumption and Production). Namely, the study demonstrates the efficient use of agricultural resources by valorizing waste seed materials (SDG 12). Moreover, the research applies green technologies to enhance sustainable industrial practices (SDG 9). Finally, by optimizing the extraction of bioactive compounds with potential health benefits, the work contributes to innovations in nutraceuticals (SDG 3).

Against this backdrop, this exploratory study represents the first comparative analysis of multiple extraction techniques and solvents used to produce bioactive extracts from both native and waste dandelion, milk thistle, and chamomile seeds. The extracted compounds were evaluated for their antioxidant, antimicrobial, and cytotoxic properties, with the goal of unlocking new commercial applications for these underutilized plant materials in a sustainable and economically viable manner. This research emphasizes the exploitation of waste seed biomass and optimizes natural product composition for diverse industries.

## 2. Materials and Methods

### 2.1. Materials

Seeds of milk thistle (*Silybum marianum*) were purchased from Prowana (Radzymin, Poland), while dandelion (*Taraxacum officinale*) and chamomile (*Chamaemelum nobile*) seeds were purchased from HerbFarm Edwin Lewczuk (Jablon, Poland) in 2021. Commercial CO_2_ (99.9%, Zaklady Azotowe “Pulawy” S.A., Puławy, Poland), absolute ethanol (min 99.9%, Chemsolute, Renningen, Germany), and distilled water (ultra-pure, TOC < 3 ppb, Aquinity membraPure GmbH, Hennigsdorf, Germany) were used for extraction processes. Phenol reagent Folin & Ciocalteu (2 M), gallic acid (97.5–102.5%), sodium carbonate (≥99.5%), quercetin (≥95%), 2,2-diphenyl-1-picryl-hydrazyl (DPPH), and iron (III) chloride (FeCl_3_, 97%) were purchased from Sigma-Aldrich (Schnelldorf, Germany) and used for evaluation of total phenolic, total flavonoid content, and radical scavenging activity of extracts. Diethyl ether (p.a., Witko, Łódź, Poland) was used for the estimation of pigment content. Isopropanol (99.8%, Chemsolute, Renningen, Germany) was used for the preparation of the extract solution before analysis.

### 2.2. Extraction from Plant Materials

The extraction of bioactive compounds from dandelion, milk thistle, and chamomile seeds was performed by supercritical fluid extraction (SFE), hot-solvent extraction using Soxhlet apparatus (SXE), and ultrasound-assisted extraction (USE). Before extractions, seeds were milled using a mill (SM100 Retsch, Katowice, Poland) with 0.5-bottom sieve mesh.

#### 2.2.1. Supercritical Carbon Dioxide Extraction from Native Seeds

The SFE process was performed in a 560 mL high-pressure unit (Micronisation unit, Sitech, Effretikon, Switzerland) described in detail elsewhere [24] using 40 g of milled plant material and sc-CO_2_ at a pressure of 45 MPa and a temperature of 40 °C during 1 h. The process conditions were optimized and detailed in our previous publications [21,22,23]. Each extraction process lasted until plant materials were exhausted.

#### 2.2.2. Extraction from Native and Waste Seeds Using a Soxhlet Apparatus

The process of extraction from 10 g of milled seeds (native and waste) was performed in an apparatus with a volume of 100 mL using 250 mL of absolute ethanol (EtOH) or ethanol/water (EtOH/H_2_O) solution (absolute ethanol to water volume ratio was 1 to 1). Processes with EtOH and EtOH/H_2_O were performed at boiling temperatures of solvents (about 78 and 85 °C, respectively). After each extraction process that lasted 3 h, solvents were evaporated from the obtained extract solution using a vacuum evaporator (Hei-VAP Precision, Heidolph, Schwabach, Germany).

#### 2.2.3. Extraction from Seeds Using an Ultrasound Bath

The process of extraction from 10 g of milled seeds was performed in a bath (Sonic 3, Polsonic, Warszawa, Poland) using 250 mL of EtOH and EtOH/H_2_O at 40 °C and 310 W for 1 h. The listed parameters were selected from the literature [5]. After each extraction process, extract solutions were separated from the plant material by gravity filtration using a cellulose filter (Round filters Rotilabo^®^ Type 112A, Ø125 mm, Carl Roth, Karlsruhe, Germany). Solvents from the obtained extract solutions were evaporated using a vacuum evaporator (Hei-VAP Precision, Heidolph, Schwabach, Germany).

The samples obtained by variation in the selection of seeds (native and waste), extraction techniques, and solvents are presented in Table 1. The resulting extraction yield was calculated as a ratio between the recovered dry samples and the initial mass of milled seeds used in the extraction processes multiplied by 100.

### 2.3. Phenolic Content in Oils and Extracts from Seeds Analysis

The samples obtained from the seeds (12.5–50.0 mg) were diluted with up to 10 mL of isopropanol. Further, the total phenolic content (TPC) in separated oils and extracts was estimated by the Folin–Ciocalteu assay as previously described [22]. All solutions were filtrated through a 0.45 μm syringe filter before analysis. Analyses were conducted in triplicate, and the results were quantified as gallic acid equivalents (GAE) per dry sample mass, utilizing a calibration curve of gallic acid in isopropanol (concentration range of 0.012–0.588 mg/mL).

### 2.4. Flavonoid Content in Oils and Extracts from Seeds Analysis

A total of 1.0 mL of each sample solution (concentration in the range of 1.25–5 mg/mL) was intensively mixed with 1.0 mL aluminum chloride solution (2% *w*/*v* in isopropanol). Furthermore, the total flavonoid content (TFC) was estimated using the aluminum chloride method as previously described [22]. All solutions were filtrated through a 0.45 μm filter before analysis. Analyses were conducted in triplicate, and the results were quantified as a mass of quercetin equivalents per dry sample mass (mg QE/g), utilizing a calibration curve of quercetin (concentration range of 0.003–0.060 mg/mL).

### 2.5. Determination of Pigments Content in Oils and Extracts from Seeds

The weighted samples (100–110 mg), after being dissolved in diethyl ether (5 mL), were placed in an ultrasonic bath for 30 min. The mixture was centrifuged for 20 min at 7000 rpm. The absorbance of supernatants was measured at 400–700 nm in a UV spectrophotometer in triplicate. Chlorophyll A showed the maximum absorbance at 662 nm, chlorophyll B at 646 nm, and total carotenoids at 470 nm. The amount of these pigments was calculated according to Dere et al. [25].

### 2.6. The Free Radical Scavenging Capacity of Oils and Extracts from Seeds

All obtained oils/extracts were dissolved in isopropanol to obtain solutions with a concentration in the range of 0.15–40 mg/mL. Further, the antioxidant activity of the samples was analyzed by the DPPH assay as previously described [22]. All solutions were filtrated through a 0.45 μm filter before analysis. Analyses were conducted in triplicate and the DPPH radical scavenging activity of the oils/extracts was expressed as IC_50_ (concentration of oil/extract that is required to inhibit 50% of the DPPH radical activity).

### 2.7. Antimicrobial Activity of Extracts from Seeds

The in vitro antimicrobial activity tests of extracts from seeds were performed using a broth microdilution method in 96-well microtitrate plates, according the recommended method of the European Committee on Antimicrobial Susceptibility Testing (EUCAST) [26] and our previously published method [22,27], allowing for the estimation of three parameters: MIC (minimum inhibitory concentration) and MBC (minimum bactericidal concentration) or MFC (minimum fungicidal concentration). These three parameters against a panel of reference microorganisms (Gram-positive, Gram-negative bacteria, and yeasts) from the American Type Culture Collection (ATCC), previously reported in [22,23], were tested. In addition, the activity of the extracts was tested on *Enterococcus faecium* ATCC 19434, *Escherichia coli* ATCC 35218, and *Candida glabrata* ATCC 15126.

### 2.8. Cytotoxic Activity of Extracts from Seeds

The cytotoxicity of selected extracts was evaluated using microculture tetrazolium test (MTT) on non-cancerous VERO (CCL-81; monkey kidney cells) and cancer-derived cells, including FaDu (HTB-43, human hypopharyngeal squamous cell carcinoma), HeLa (CCL-2, human cervical adenocarcinoma), and RKO (CRL-2577, human colon cancer), following a previously described methodology [28]. Stock solutions (100 mg/mL in DMSO) of tested extracts were serially diluted in cell media and incubated with a selected cell line for 24 h. Subsequently, cellular viability was assessed—(3-(4,5-dimethylthiazol-2-yl)-2,5-diphenyltetrazolium bromide) diluted in cell media was added and after 3 h, the amount of formazan product was measured spectrophotometrically at 540 and 620 nm using the Synergy H1 Multi-Mode Microplate Reader (BioTek Instruments, Inc. Winooski, VT, USA) with Gen5 software (ver. 3.09.07; BioTek Instruments, Inc.) and the results were exported to GraphPad Prism software (version 9.0) for further analysis. Finally, the CC_50_ (concentration resulting in a 50% reduction in cell viability) values were calculated from dose–response curves (non-linear regression).

### 2.9. Statistical Analysis

The results of the current study are presented with mean ± standard deviation; a one-way ANOVA with a post hoc Tukey HSD (Honestly Significant Difference) test was used to evaluate the significant differences in the extraction yields, the content of phenolics, flavonoids, and pigments, as well as the antioxidant activity of extracts from native and defatted seeds (the number of compared samples for each plant was 7). Statistical analyses for these results were performed using the Astatsa online statistical calculator [29], and *p*-values lower than 0.05 are reported as “significant” while *p*-values higher than 0.05 are reported as “not significant”. The differences in the CC_50_ values between the four cell lines for the cytotoxic activity of the selected extracts were statistically determined using GraphPad Prism (two-way ANOVA, Tukey’s multiple comparisons test).

## 3. Results

### 3.1. Extraction from Native and Waste Dandelion, Milk Thistle, and Chamomile Seeds

**Figure 1:**
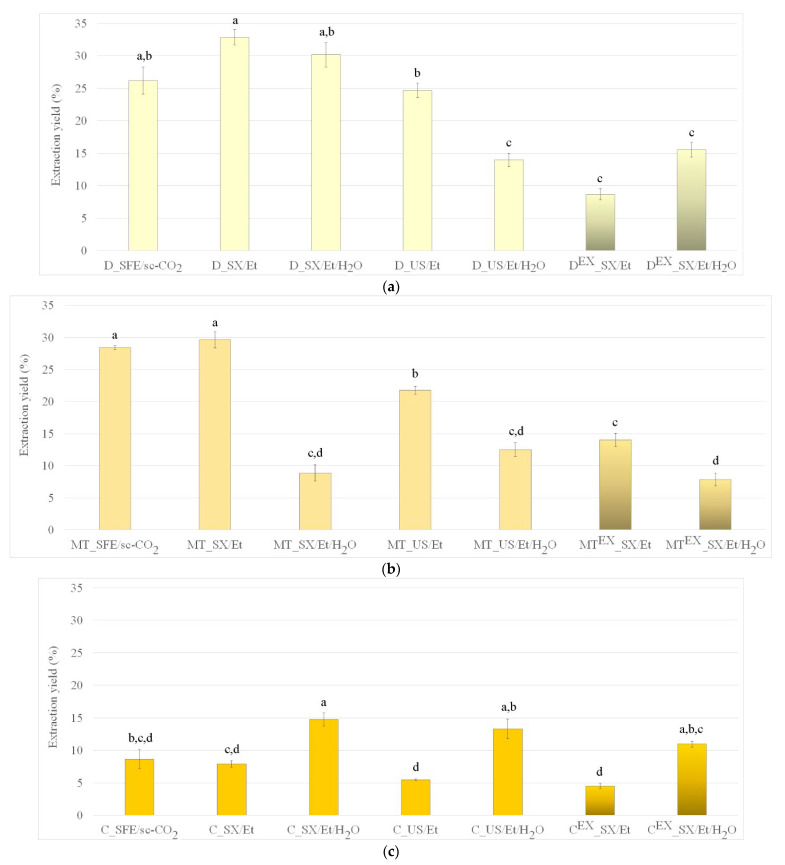
Extraction yield for native and waste seeds of (**a**) dandelion, (**b**) milk thistle, and (**c**) chamomile obtained by SFE, SXE, and USE using sc-CO_2_, absolute ethanol, or aqueous ethanol as solvents. Different letters (a–d) suggest that values are significantly different (*p* < 0.05).

Native seeds of dandelion, milk thistle, and chamomile were extracted by SFE, SXE, and USE techniques using sc-CO_2_, absolute ethanol, or ethanol/water mixture. Results expressed as extraction yield are shown in Figure 1. Additional data from replicate experiments can be cross-referenced from the Supplementary Materials for completeness (Table S1) while calculated *p*-values can be seen in Table S2 in detail.

The SFE process enabled the recovery of relatively high amounts of oil from native dandelion, milk thistle, and chamomile seeds, achieving yields of 26.2 ± 2.1, 28.4 ± 0.3, and 8.7 ± 1.5%, respectively. Compared to SFE yields (milk thistle > dandelion > chamomile), results for SXE and USE techniques in the current study varied depending on both the technique and solvent used. SXE with absolute ethanol resulted in extraction yields that decreased in the following order: dandelion > milk thistle > chamomile. Namely, while the changes were statistically insignificant (*p* > 0.05), a higher amount of recovered extracts was recorded for dandelion and milk thistle (32.9 ± 1.2 and 29.6 ± 1.3%, respectively) with a decreased amount of recovered extract from chamomile seeds (7.9 ± 0.5%). On the other hand, SXE with aqueous ethanol insignificantly increased the amount of separated extract from dandelion to 30.2 ± 1.9%, significantly increased (*p* < 0.05) the amount of separated extract from chamomile to 14.8 ± 1.0%, and significantly decreased (*p* < 0.01) the amount of separated extract from milk thistle seeds to 8.9 ± 1.2% compared to SFE. In this case, yields decreased in following order: dandelion > chamomile > milk thistle. Standard deviations between SFE and SXE with aqueous ethanol for milk thistle are unusually high, which confirms the variability in experimental conditions.

The utilization of USE with absolute ethanol for the extraction from native seeds decreased extraction yields compared to SXE (yields decreased in the same order as for SXE). The reason for these decreases could be found in the lower temperature employed for the USE process. Namely, higher temperatures used for SXE increase the diffusion of extractable compounds improving mass transfer [30].

SFE primarily facilitated the isolation of non-polar oils from the seeds, aligning with the non-polar nature of sc-CO_2_. In contrast, SXE and USE, when performed with the polar solvents absolute ethanol and aqueous ethanol, enabled the recovery of both liquid and solid extract fractions from the seeds. The choice of solvent significantly impacted the extraction efficiency and composition of the extracts, demonstrating the importance of solvent polarity in targeting specific bioactive compounds. Interestingly, the use of aqueous ethanol in SXE and USE resulted in a decreased recovery of extracts from dandelion and milk thistle seeds compared to absolute ethanol. This suggests that the addition of water likely limited the solubility of certain hydrophobic compounds present in these seeds, reducing the overall yield. In contrast, chamomile seeds showed an increase in extract recovery with aqueous ethanol, which may be attributed to the higher content of hydrophilic, water-soluble polysaccharides and proteins [31]. These findings suggest that chamomile seeds contain more water-soluble bioactive compounds, which are more efficiently extracted using a solvent mixture with higher polarity. This divergence in extract recovery demonstrates that solvent selection plays a critical role in optimizing the extraction of bioactive compounds and highlights the necessity of tailoring extraction methods based on the specific chemical composition of each plant seed. Additionally, using green solvents like aqueous ethanol aligns with the principles of sustainable chemistry, as it reduces the reliance on pure organic solvents while enhancing the extraction of hydrophilic compounds, further promoting eco-friendly extraction processes.

The obtained yields from native seeds (Figure 1) using SFE with neat sc-CO_2_ and using SXE and USE with absolute ethanol are higher compared to the results reported in the literature for other solvents. For instance, it was reported that the extraction yield of 22.4% was obtained by SXE with hexane from dandelion seeds [16] and that a yield of 16.1% was obtained from milk thistle seeds using USE with hexane [32].

As SXE enabled higher yields, further extractions from seeds remained after SFE for waste valorization was performed with the SXE technique using both ethanol and ethanol/water as solvents. It can be seen (Figure 1) that after the removal of oil using sc-CO_2_, the remaining waste seeds contain a significant number of extractable compounds. Waste seeds gave extract yields in the range of 4.5 ± 0.4 to 15.5 ± 1.1%. The yields of extracts collected by absolute ethanol as the solvent decreased in the following order: milk thistle > dandelion > chamomile. The addition of water to ethanol for SXE had a similar effect for extraction from waste seeds as for extraction from native seeds (the order of yields was the same: dandelion > chamomile > milk thistle). Aqueous ethanol led to a statistically significant decrease (*p* < 0.05) in extract isolation from waste milk thistle seeds (Figure 1b), while it resulted in a statistically insignificant increase (*p* > 0.05) in extract isolation from dandelion (Figure 1a) and a statistically significant increase (*p* < 0.05) in extraction yields for chamomile (Figure 1c).

### 3.2. Total Phenolics and Flavonoids Content in Oils and Extracts from Native and Waste Dandelion, Milk Thistle, and Chamomile Seeds

**Figure 2:**
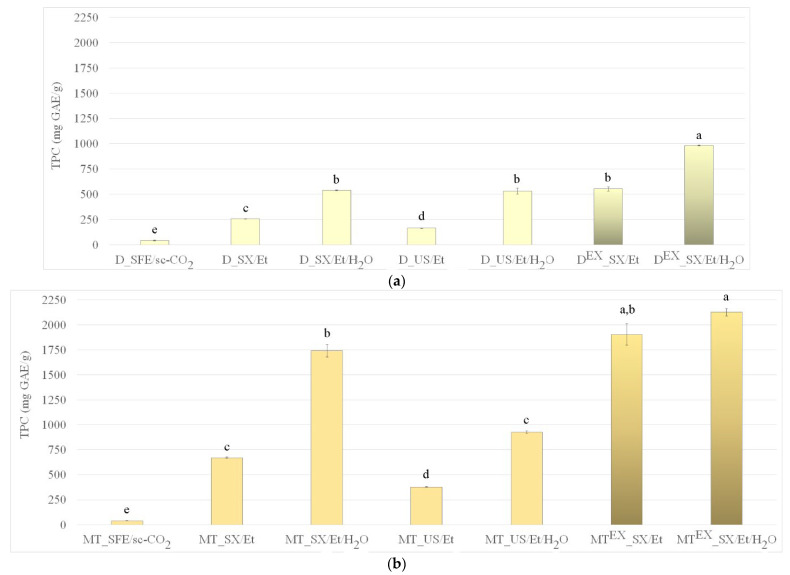

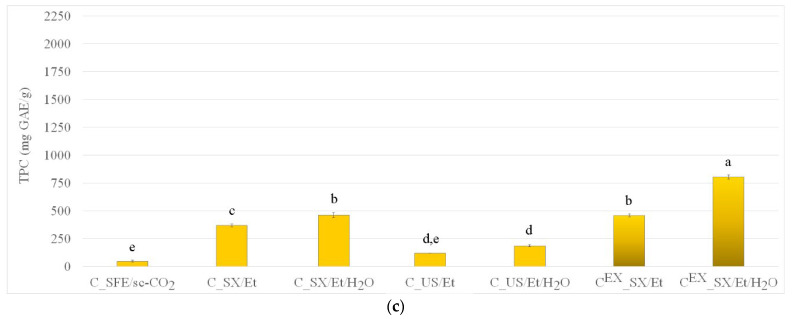
Total phenolic content in oils and extracts from (**a**) dandelion, (**b**) milk thistle, and (**c**) chamomile native and waste seeds obtained via SFE, SXE, and USE using sc-CO_2_, absolute ethanol, or aqueous ethanol as solvents. Different letters (a–e) suggest that values are significantly different (*p* < 0.05).

The TPC detected in oils (samples obtained by SFE) and extracts (samples obtained by conventional extractions) are presented in Figure 2. Additional data from replicate experiments can be cross-referenced from the Supplementary Materials for completeness (Table S3) while calculated *p*-values can be seen in Table S4 in detail. The oils isolated from all tested seeds by SFE contained relatively low concentrations of phenolic compounds (up to 37.9 mg GAE/g). This observation can be attributed to the non-polar nature of sc-CO_2_ that favors the extraction of non-polar substances like oils, but it is limited in its ability to recover more polar bioactive compounds such as phenolics.

To enhance the recovery of phenolics, conventional extraction techniques using polar solvents (absolute and aqueous ethanol) were employed. These solvents exhibited a greater affinity for polar compounds located in the seed matrices, leading to significantly higher TPC in the resulting extracts compared to oils obtained by sc-CO_2_. This demonstrates the importance of selecting appropriate extraction methods and solvents based on the target compounds’ chemical properties. Accordingly, it was also shown that the inclusion of water in the ethanol solvent system for both SXE and USE significantly enhanced the extraction of phenolics across all tested seeds (*p* < 0.05), with the exception of chamomile extracts obtained via USE, where the increase in TPC was statistically insignificant. Ethanol effectively disrupts the interactions between phenolic compounds and plant matrices, allowing for easier extraction, while water promotes the swelling of plant cells, which in addition facilitates the diffusion of these compounds [30]. However, pure water is generally ineffective as a solvent for phenolics, as many phenolic compounds exhibit higher solubility in less polar organic solvents [33]. In contrast, binary solvent systems (such as EtOH/H_2_O) provide a synergistic effect, yielding higher concentrations of phenolic and flavonoid compounds in extracts compared to using mono-solvent systems like absolute ethanol or water alone [30,33]. For instance, the substantial increase in phenolic content in milk thistle extracts evident in the TPC spike observed in Figure 2b (regardless of a decrease in extraction yield seen in Figure 1b) is likely a result of the selective solubility of phenolic compounds in the aqueous ethanol used during SXE and USE. Namely, as Drouet et al. [5] noted, the aqueous ethanol (especially 50% concentration) was optimal for extracting silymarin, a key phenolic compound found in milk thistle.

Selected extraction techniques also had a significant influence on TPC. Although the USE technique led to a significantly higher amount (*p* < 0.05) of recovered phenolics compared to SFE (except for chamomile when using USE with absolute ethanol, which is not significantly different from SFE), it led to a lower amount of phenolics compared to SXE. The elevated temperatures used during SXE can improve the permeability of plant cell walls and break the interactions between phenolic compounds and macromolecules, such as proteins and polysaccharides. This process facilitates the release and recovery of phenolic compounds into the extracts [30]. The thermal energy helps to disrupt complex bonds, increasing the solubility of phenolics, which are typically bound within the plant matrix. The highest values of TPC in all the tested native seeds were recorded when SXE with aqueous ethanol was used, especially for milk thistle seeds when TPC reached a value of 1741.8 ± 61.2 mg GAE/g (Figure 2b). In contrast to the findings of this study, previous research indicated that USE achieved a higher TPC for sample extracted from mahua seeds compared to SXE [11]. The superior performance of USE in certain studies can be attributed to its ability to cause cavitation, where the rapid formation and collapse of bubbles generates localized pressure, disrupting plant cell walls. This process enhances solvent penetration into the plant matrix and accelerates the mass transfer of bioactive compounds, which theoretically should result in a more efficient extraction process, especially for thermally sensitive compounds that may degrade under the higher temperatures used in SXE. On the other hand, those results could be attributed to solvent selection (i.e., hexane) and prolonged SXE process that lasted 8 h [11]. The differing results between the current study and previous research underscore the importance of plant material type, specific compound interactions, and process conditions. While SXE proves effective for phenolic recovery in the seeds studied here, USE offers advantages in cases where milder conditions are necessary for preserving delicate compounds.

There is a large number of articles in the available literature on the topic of extraction from native milk thistle seeds and TPC in those extracts in contrast to scarce information on TPC in extracts obtained from native dandelion and especially chamomile seeds. On the other hand, there is a lack of information on TPC in extracts obtained from waste or defatted seeds. The list of literature TPC data for relevant plant material sources can be seen in Table S5 [7,14,16,21,22,23,31,34,35,36,37,38,39,40,41,42,43,44,45,46]. By comparison, the values of TPC recorded in the current study are considerably higher. The highest TPC values recorded for the extracts from waste seeds from dandelion, milk thistle, and chamomile in the current study were 984.1 ± 4.1, 2125.8 ± 37.4, and 803.9 ± 19.5 mg GAE/g, respectively. On the other hand, the literature reports TPC in an aqueous methanol extract from dandelion petals to be 253.1 mg/g [7], in milk thistle seed extracts obtained by a ternary mixture of solvents composed of CO_2_/EtOH/H_2_O to range from 237 to 758 mg GAE/g [34], and in aqueous extracts from chamomile seeds to range from 16.4 to 22.4 mg GAE/g [31]. The presented results also showed higher TPC values detected in extracts from the waste seeds of dandelion, milk thistle, and chamomile compared to other literature reports for waste seeds. For instance, a TPC value up to 144 mg GAE/g was detected in extracts from defatted pitaya seeds [30]; 48.9 mg GAE/g was detected in extracts from defatted evening-primrose seeds [46] and a value of up to 3.2 mg GAE/g was detected in extracts from defatted canola seeds [47].

**Figure 3:**
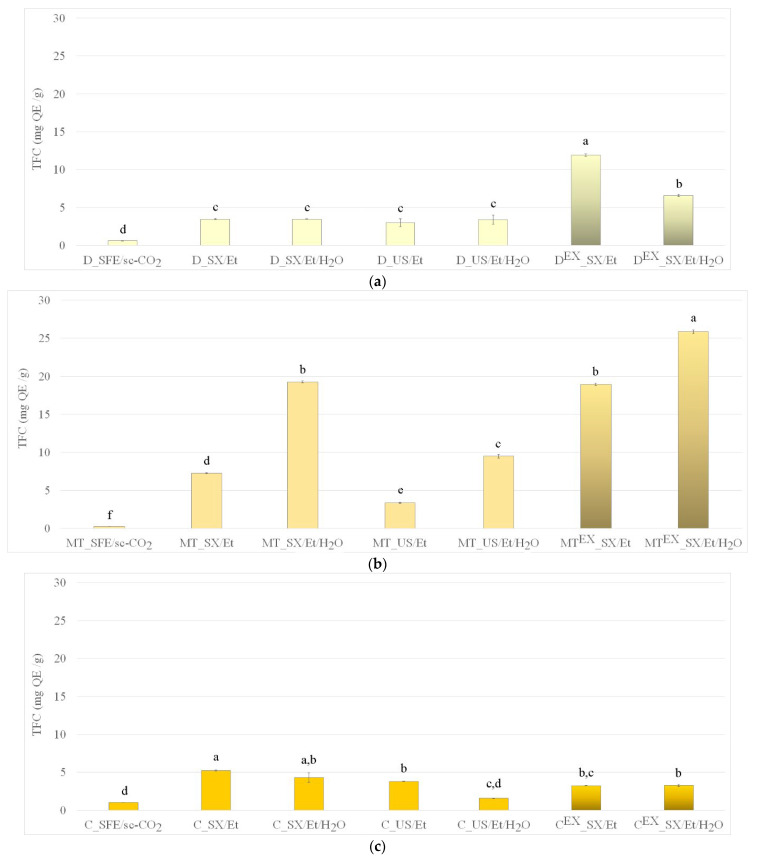
Total flavonoid content in oils and extracts from (**a**) dandelion, (**b**) milk thistle, and (**c**) chamomile native and waste seeds obtained by SFE, SXE, and USE using sc-CO_2_, absolute ethanol, or aqueous ethanol as solvents. Different letters (a–f) suggest that values are significantly different (*p* < 0.05).

The influence of extraction parameters on flavonoids content in extracts can be seen in Figure 3. Additional data from replicate experiments can be cross-referenced from the Supplementary Materials for completeness (Table S6), while calculated *p*-values can be seen in Table S7 in detail. The extraction of flavonoids followed a similar trend to the extraction of phenolics. Standard deviations between oils obtained by SFE and extracts obtained by other techniques are statistically significantly high (*p* < 0.01), which confirms the variability in the experimental conditions and the samples’ heterogeneity. The only exception was the chamomile sample C_US/Et/H_2_O that contained an insignificantly higher amount of flavonoids compared to the C_SFE/sc-CO_2_ sample (*p* > 0.05).

As for the native seeds, the lowest and the highest TFC value was detected for the milk thistle oil obtained by SFE (0.2 ± 0.0 mg QE/g) and for its extract obtained by SXE with aqueous ethanol (19.3 ± 10.0 mg QE/g), respectively. As for the waste seeds, the lowest TFC value was detected in chamomile extracts (3.2 ± 0.0 mg QE/g) and the highest in milk thistle extracts obtained using aqueous ethanol (25.8 ± 0.2 mg QE/g). A comparison of the recorded TFC data with the literature reports shown in Table S8 [16,21,22,23,31,41,48,49,50,51,52,53,54,55,56,57,58,59,60] indicates the superiority of the current results. The presented results also showed higher values of TFC detected in the waste material of dandelion, milk thistle, and chamomile compared to literature reports for different waste seeds. For instance, it was reported that extracts from defatted pitaya seeds contained up to 10.7 mg QE/g [30] and extracts from waste date seeds contained 8.5 mg QE/g TFC [61].

The findings of the present study support the use of the extraction techniques presented for producing extracts rich in TPC and TFC from various seeds of the *Asteraceae* family. Phenolic and flavonoid compounds are well known for their diverse physiological activities, including antimicrobial, antioxidant, anti-inflammatory, antitumor, cardiovascular, and neuroprotective effects [30,62,63]. Due to their significant health benefits, products rich in phenolics and flavonoids (such as foods, supplements, and cosmetics) are increasingly preferred by consumers [63].

### 3.3. Pigment Content in Samples from Native and Waste Milk Thistle, Dandelion, and Chamomile Seeds

**Figure 4:**
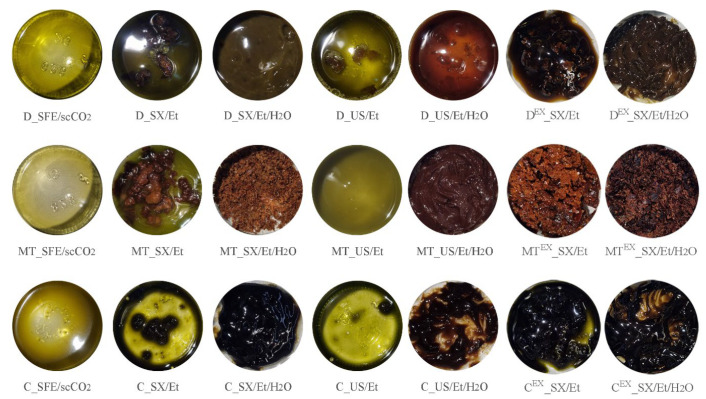
Images of samples from native and waste seeds of dandelion (D), milk thistle (MT), and chamomile (C) obtained by SFE, SXE, and USE using sc-CO_2_, absolute ethanol, or aqueous ethanol as solvents.

Images of samples from seeds are shown in Figure 4 (the mass of extracts ranged from 0.5 to 3.0 g while the diameter of the glass jar was 3 cm).

It can be observed that the color of oils isolated by SFE varied from dark yellow (for milk thistle) to dark olive-green (for dandelion). On the other hand, when the technique was changed to SXE and USE, the color and consistency of the extracts also changed. Moreover, the addition of water to ethanol for extractions further darkened the extracts, resulting in dark brown and semi-solid or solid extracts.

The change in the color of the dandelion, milk thistle, and chamomile oil/extracts resulted from variations in pigment recovery, as evident in Table 2. Data from replicate experiments for chlorophyll A can be cross-referenced from the Supplementary Materials for completeness (Table S9), while calculated *p*-values can be seen in Table S10 in detail. Higher temperatures usually lead to a degradation of pigments [64] which was not the case for extracts from tested seeds obtained using absolute ethanol. In all cases, the use of absolute ethanol as a solvent resulted in higher amounts of recovered chlorophyll A from the tested seeds compared to sc-CO_2_ and aqueous ethanol solvents. However, for extracts from native dandelion seeds, this increase in chlorophyll A isolation was not statistically significant (*p* > 0.05). On the other hand, there is no clear trend in the isolation of chlorophyll B concerning the choice of solvent for conventional extractions; however, the lowest values were consistently obtained using sc-CO_2_. In terms of carotenoid isolation, aqueous ethanol yielded the lowest amounts.

Overall, the highest number of recovered pigments from both native and waste seeds was detected in extracts from chamomile. The content of chlorophyll A, chlorophyll B, and carotenoids reached up to 805.4 ± 3.2 mg/kg, 373.3 ± 0.6 mg/kg, and 178.6 ± 0.7 mg/kg, respectively. The values reported in Table 2 are significantly higher compared to commercially available oils and extracts from common food sources. For instance, chlorophyll and carotenoid contents in virgin olive oils were in the range 0.2–62.0 mg/kg and 0.5–31.5 mg/kg, respectively [65], while chlorophyll A and chlorophyll B contents in pistachio extracts were in the range 2.0–7.8 mg/kg and 0.5–3.3 mg/kg, respectively [66]. Given the numerous health benefits associated with the pigments evaluated in this study [64], their relatively high concentrations found in oils and extracts from dandelion, milk thistle, and chamomile seeds highlight the potential of these natural products for use in food products and dietary supplements. Moreover, the extraction methods employed were optimized not only for yield but also to minimize environmental impact, aligning with green chemistry principles. This comprehensive approach reinforces the relevance of these extracts as eco-friendly, high-potential ingredients, warranting further investigation for broader applications without compromising environmental integrity.

### 3.4. Antioxidant Activity of Samples from Waste and Native Milk Thistle, Dandelion, and Chamomile Seeds

Oils recovered from dandelion, milk thistle, and chamomile by the SFE technique provided the lowest antioxidant activity with IC_50_ of 51.0 ± 0.1, 30.9 ± 0.9, and 17.6 ± 0.5 mg/mL, respectively (Table 2). Data from replicate experiments can be cross-referenced from the Supplementary Materials for completeness (Table S11) while the calculated *p*-values can be seen in Table S12 in detail. The results indicate that the utilization of SXE and USE enabled the production of extracts with significantly stronger antioxidant activity (*p* < 0.01) compared to SFE. These high standard deviations confirm the high influence of experimental conditions that lead to the heterogeneity of samples. Moreover, SXE and USE techniques gave comparable results for DPPH radical scavenging activity with an IC_50_ that ranged from 0.3 to 3.3 mg/mL. The use of aqueous ethanol instead of absolute ethanol led to the recovery of extracts by SXE, which leads to an increase in antioxidant activity that was statistically insignificant (*p* > 0.05) for all native seeds, while in the case of USE, an increase in antioxidant activity was statistically significant (*p* < 0.01) for dandelion and milk thistle. An increase in antioxidant activity with a decrease in ethanol concentration was also previously reported for extracts from defatted pitaya seeds [30].

Although high antioxidant activity is usually connected with TPC or TFC [33], this was not the case for the extracts obtained in the current study. For instance, the milk thistle extract MT_SX/Et/H_2_O that had a TPC of 1741.8 ± 61.2 mg GAE/g and TFC of 19.3 ± 0.1 mg QE/g provided IC_50_ with 0.4 ± 0.0 mg/mL. On the other hand, the dandelion sample D_US/Et/H_2_O with a lower TPC of 532.4 ± 30.8 mg GAE/g and a lower TFC of 3.4 ± 0.6 mg QE/g provided stronger antioxidant activity with an IC_50_ of 0.3 ± 0.1 mg/mL. While this difference in DPPH radical scavenging activity might appear small on an absolute scale, it is important to point out that the dandelion extract, despite having approximately 3-fold and 5-fold lower TPC and TFC, respectively, demonstrated comparable or even stronger antioxidant activity. This suggests that the phenolic and flavonoid compounds in the dandelion extract are particularly potent or that other bioactive compounds present in extracts act synergistically and contribute to its potent antioxidant capacity. Moreover, extracts obtained from chamomile seeds showed stronger antioxidant activity compared to extracts from the other two tested seeds, regardless of the lowest TPC and TFC. The correlation of TPC and TFC with DPPH radical scavenging activity was also irregular for the extracts from the different plant materials reported in the literature. Namely, it was reported that the DPPH radical inhibition induced by barley malt extracts has a strong correlation with flavonoid content and a weak correlation with TPC [33]. The inconsistency between TPC values and antioxidant activity was also reported for extracts obtained from waste sesame seeds [67].

Overall, the recorded results indicated that extracts from dandelion, milk thistle, and chamomile seeds might play a potential role as health-promoting antioxidant agents in human diets for the pharmaceutical industry. This antioxidant activity was more profound compared to previous reports for oils/extracts from mahua seeds (the best IC_50_ value was 106.6 mg/mL obtained by the USE technique with hexane) [11] and cowherb seeds (the best IC_50_ value was 0.5 mg/mL obtained by microwave-assisted extraction with methanol) [68]. In addition, previous reports on the antioxidant activity of water and acetone extracts from chamomile flowers reported IC_50_ values in the range from 0.59 to 37.6 mg/mL [42].

There is no clear trend in the amount and composition of oils and extracts among the three *Asteraceae* family plants tested. However, the highest yield of extracts was obtained from both native and waste dandelion seeds. Extracts from both native and waste milk thistle seeds exhibited the highest phenolic and flavonoid content. The highest concentration of chlorophylls and carotenoids was found in extracts from both native and waste chamomile seeds. Notably, extracts obtained by SXE with aqueous ethanol from all three plants (dandelion, milk thistle, and chamomile native and waste seeds) demonstrated comparable DPPH radical scavenging activity. Moreover, these extracts contained high level of phenolic, flavonoid, and pigment compounds. Numerous studies have reported on the significant health benefits that these compounds exhibit. Consequently, phenolic-, flavonoid-, and pigment-rich extracts from dandelion, milk thistle, and chamomile seeds have been selected for further antimicrobial and cytotoxic testing.

### 3.5. Antimicrobial Activity of Extracts from Milk Thistle, Dandelion, and Chamomile Seeds

**Figure 5:**
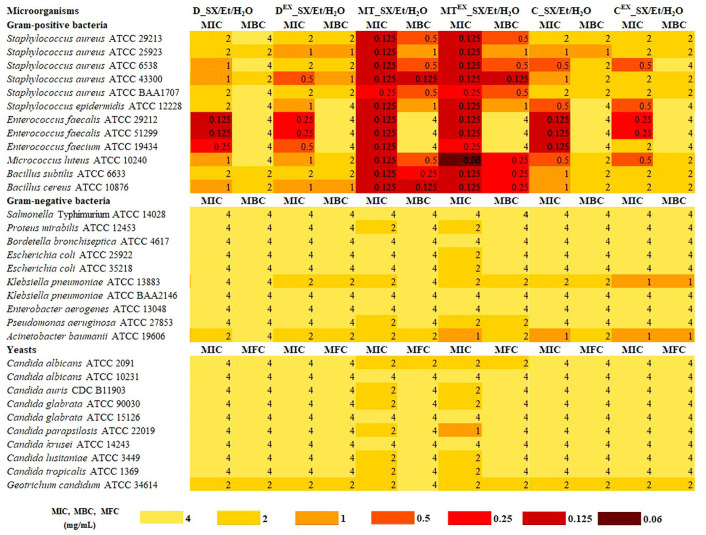
Antimicrobial data (MIC—minimum inhibitory concentration, MBC—minimum bactericidal concentration, and MFC—minimum fungicidal concentration) for extracts from dandelion (D), milk thistle (MT), and chamomile (C) seeds obtained by SXE using aqueous ethanol against reference microorganisms.

The antibacterial abilities of the extracts from native and waste milk thistle, dandelion, and chamomile seeds are shown in Figure 5. These extracts showed varying activity against all microorganisms, with MIC values ranging from 0.06 to 4 mg/mL. MIC values suggested that tested Gram-positive bacteria were more sensitive to extracts than yeasts and Gram-negative bacteria strains. In all of the cases, the MIC values for Gram-negative bacteria were higher (1–4 mg/mL) in comparison to the MIC values for Gram-positives (0.06–2 mg/mL). Indeed, with the exception of *K. pneumoniae* ATCC 13883 and *A. baumanii* ATCC 19606, all of the Gram-negative strains were able to grow at relatively high extract concentrations. Nonetheless, articles from the literature reported inferior MIC values against Gram-negative bacteria. For instance, it was reported that milk thistle extracts do not show any action against *E. coli* [69], chamomile oil shows MIC > 10 mg/mL against *K. pneumonia*, and dandelion oil shows MIC of 16 mg/mL against *P. aeruginosa* [16]. The best inhibitory activity against Gram-positive bacterial strains was observed for the sample MT^EX^_SX/Et/H_2_O with MIC values in the range from 0.06 to 0.25 mg/mL. The sample MT_SX/Et/H_2_O obtained from the same plant (milk thistle) was followed with MIC in the range 0.125–0.25 mg/mL. The highest susceptibility among bacteria was found for *M. luteus* ATCC 10240 with MIC values of 0.06 mg/mL and 0.125 mg/mL for the extracts from native and waste milk thistle seeds, respectively. These extracts were also effective against *Staphylococcus* spp. at the lowest concentration analyzed (0.06–0.25 mg/mL), showing bactericidal activity. In the case of two methicillin-resistant *S. aureus* strains (MRSA) (ATCC 43300 and ATCC BAA-1707), extracts from milk thistle (MT_SX/Et/H_2_O and MT^EX^_SX/Et/H_2_O) showed good activity (0.125–0.25 mg/mL). These extracts also showed bactericidal activity. Previous reports on antibacterial activity against MRSA for methanolic milk thistle extracts stated MIC values of 7.8 and 15.6 mg/mL [69] and for the ethanolic milk thistle extracts a value of 2.3 mg/mL [70]. Extracts from chamomile and dandelion showed slightly weaker activity against the tested microorganisms. These extracts showed weak activity with MIC values in the range of 0.5–2 mg/mL against two MRSA (ATCC 43300 and ATCC BAA-1707). Interestingly, extracts from the native seeds (C_SX/Et/H_2_O and D_SX/Et/H_2_O) exhibited MIC values in the range of 0.125–2 mg/mL, while extracts from waste seeds (C^EX^_SX/Et/H_2_O and D^EX^_SX/Et/H_2_O) showed slightly weaker activity (MIC = 0.25–2 mg/mL). The extracts from chamomile seeds showed moderate activity (MIC = 0.5 mg/mL) against *M. luteus* ATCC 10240, *S. aureus* ATCC 6538, and *S. epidermidis* ATCC 12228. Our initial tests for chamomile and dandelion oils obtained by neat sc-CO_2_ showed inferior activity against *Staphylococcus* bacteria with MIC values in the range of 1.25–5 mg/mL [23] and 2–4 mg/mL [16], respectively.

All extracts showed very good activity against *E. faecalis* ATCC 29212 and *E. faecalis* ATCC 51299 with MIC = 0.125–0.25 mg/mL. A good activity (MIC = 0.125–0.5 mg/mL) in all extracts (except C^EX^_SX/Et/H_2_O) was also shown against *E. faecium* ATCC 19434. On the other hand, our initial studies for chamomile and dandelion oils obtained by neat sc-CO_2_ showed MIC values of 5 mg/mL [23] and 4 mg/mL [16] against *Enterococcus* bacteria, respectively. The tests for evaluating the antifungal potency of extracts showed no appreciable antifungal activity. The best inhibitory activity was observed for MT^EX^_SX/Et/H_2_O against *Candida parapsilosis* with MIC = 1 mg/mL, while no marked difference was observed in the activity profiles for other extracts (MIC = 2–4 mg/mL). However, articles from the literature reported that methanolic extracts from milk thistle inhibit *Candida* only with MIC values in the range from 125 to 250 mg/mL [69]. Considering that the literature reports on dandelion, milk thistle, and chamomile seeds show MIC values in the range of 1.1–250 mg/mL [16,23,69,70], it can be inferred that the extracts obtained in the current study are superior and that the presented techniques for production improve their antimicrobial activity.

### 3.6. Cytotoxic Activity of Extracts from Milk Thistle, Dandelion, and Chamomile Seeds

The CC_50_ values recorded for selected seed extracts, evaluated on non-cancerous monkey kidney (VERO), human hypopharyngeal squamous cell carcinoma (FaDu), human cervical adenocarcinoma (HeLa), and human colon cancer (RKO) cells, are presented in Table 3. The evaluation of the influence of dandelion, milk thistle, and chamomile seed extracts on cell lines revealed low cytotoxic potential (200 µg/mL < CC_50_ ≤ 500 µg/mL) [71,72] for most of the tested extracts. Only the milk thistle extracts, MT^EX^_SX/Et/H_2_O and MT_SX/Et/H_2_O, were moderately cytotoxic to FaDu cancer cells. Both extracts from dandelion were not cytotoxic to non-cancerous cells, while extracts from native dandelion and native chamomile seeds showed no cytotoxicity towards HeLa.

No cytotoxicity was also found for all dandelion and chamomile extracts on RKO cells. On the other hand, extracts tested in the current study showed stronger cytotoxic activity against FaDu, HeLa, or RKO cells compared to oils obtained from dandelion and chamomile by the neat sc-CO_2_ as previously reported [22,23]. Moreover, the activity of tested extracts was comparable or stronger compared to the literature. For instance, it was reported that methanolic extracts from African tulip tree leaves express CC_50_ of 358, 914, and 382 µg/mL against FaDu, HeLa, and RKO cells, respectively [73] and that methanolic extract from truffles shows CC_50_ in the range of 148–300 µg/mL against HeLa cells [74].

**Figure 6:**
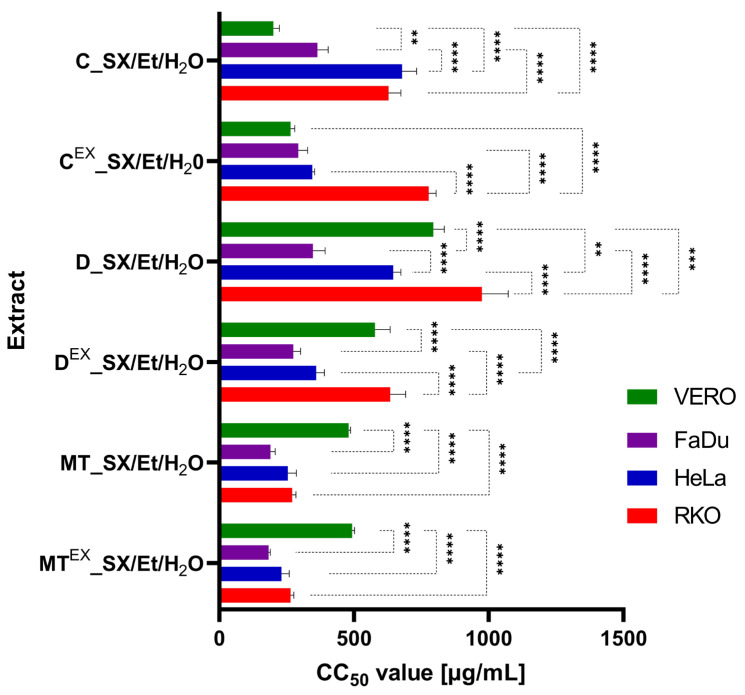
Cytotoxicity of selected extracts from dandelion (D), milk thistle (MT), and chamomile (C) seeds obtained by SXE using aqueous ethanol. (CC_50_—concentration resulting in a 50% reduction in cell viability; ** *p* < 0.01; *** *p* < 0.001; **** *p* < 0.0001).

The SI indexes ranging between 1.87 and 2.7 shown in Table 3 lead to the conclusion that the extract from waste milk thistle seeds shows selective anticancer cytotoxicity against all tested cancer cells. In the case of both milk thistle extracts, there were no statistically significant differences (*p* > 0.05) between their cytotoxic influence on different cancer cell lines (Figure 6). However, they were significantly less cytotoxic (*p* < 0.0001) to non-cancerous cells compared to cancer cell lines. Table S13 presents the coefficient of variation (CV) calculated for the cytotoxicity results, ranging for samples tested on VERO cells from 1.31% to 11.39%. Interestingly, for native dandelion seed extracts, significant differences in the cytotoxicity to all tested cell lines were observed, and for waste dandelion seed extract, there were no significant differences between VERO and RKO, as well as between FaDu and HeLa. When the cytotoxicity of native and waste milk thistle extracts was compared on the same cell line, there were no significant differences (*p* > 0.05). Both chamomile extracts showed similar cytotoxicity on VERO and FaDu but showed differences on HeLa (*p* < 0.0001) and RKO (*p* < 0.001). However, the dandelion extracts’ cytotoxicity was different (*p* < 0.0001) on VERO, HeLa, and RKO, but comparable on FaDu cells. Overall, the differences in cytotoxicity between the extracts from native and waste seeds seem to be dependent both on the tested plant material for sample preparation and the type of tested cells.

The findings of this study highlight a dual-pathway utilization strategy for dandelion, milk thistle, and chamomile seeds, with implications for sustainable industry practices. The oils obtained from the native seeds via SFE present high-yield, solvent-free products, ideal for use as edible oils in the food industry. These oils align with consumer demand for natural and minimally processed products while retaining nutritional value, making them suitable for culinary purposes or as functional ingredients in health-promoting food formulations. The high yield that was achieved also enhances their industrial feasibility, supporting sustainable food production. Meanwhile, the remaining waste seeds, rich in bioactive compounds like phenolics and flavonoids, offer significant potential for high-value applications in the pharmaceutical, nutraceutical, or cosmetics industries. The bioactivity observed in waste seed extracts, including potent antioxidant, antimicrobial, and selective anticancer properties, positions these byproducts as valuable candidates for therapeutic formulations, dietary supplements, or natural preservatives. This dual utilization approach underscores the importance of integrating waste valorization into production pipelines, minimizing resource wastage, and promoting a circular economy. By adopting such strategies, industries can leverage both the edible oils and bioactive extracts from these seeds, aligning with sustainability goals such as the United Nations’ Sustainable Development Goals (SDG 12: Responsible Consumption and Production). This model supports eco-friendly innovation while maximizing the commercial and environmental value of plant-based resources.

## 4. Conclusions

The results of the present study demonstrated that substantial amounts of solvent-free and biologically valuable oils (up to 281 mg per g of seeds) could be effectively extracted from dandelion, milk thistle, and chamomile seeds using the environmentally friendly method of supercritical fluid extraction. Notably, following the initial oil extraction using non-polar sc-CO_2_, this study revealed for the first time that up to 142 mg of additional extracts per 1 g of waste seeds can be further obtained using ethanol in conventional extraction methods. Dandelion seeds yielded the highest quantities of both oil and extracts.

The phenolic, flavonoid, and pigment content in the produced samples exceeded levels reported in the existing literature. Specifically, extracts from waste milk thistle seeds contained the highest levels of phenolic (2125.8 mg GAE/g) and flavonoid (25.8 mg QE/g) compounds. The highest amounts of chlorophyll A (805.4 mg/kg), chlorophyll B (373.3 mg/kg), and carotenoids (178.6 mg/kg) were detected in extracts from waste chamomile seeds.

While oils and extracts from native seeds contained significant amounts of bioactive compounds, extracts from waste seeds demonstrated stronger biological activity. The minimal extract concentrations required to inhibit bacteria and yeast growth ranged from 0.06 to 4 mg/mL. Furthermore, the extract from waste milk thistle seeds showed selective anticancer cytotoxicity against all tested cancer cells, with a selectivity index in the range of 1.9–2.7. The observed differences in cytotoxicity between extracts from native and waste seeds varied depending on the plant source and the type of cultured cells.

The presented findings highlighted the considerable potential of dandelion, milk thistle, and chamomile seeds as sources of bioactive compounds. By employing optimized, sustainable extraction techniques (such as supercritical fluid extraction and ethanol-based extractions), this study highlights the feasibility of eco-friendly recovery processes that align with green chemistry principles. Oils recovered from native seeds show promise for food industry applications, while extracts recovered from waste seeds of dandelion, milk thistle, and chamomile are particularly suited for pharmaceutical applications. This research provides valuable insights into the sustainable utilization of both native and waste seeds, supporting the development of green extraction technologies for bioactive compound recovery.

Given the promising bioactivity of the extracts, the next step is to perform clinical trials to assess the safety and efficacy of the produced oils and extracts for human consumption. In addition, the scalability of the extraction methods used should be evaluated to assess their potential for cost-effective and efficient large-scale production in the food, pharmaceutical, and nutraceutical industries while promoting sustainable practices and contributing to the circular economy by minimizing waste and maximizing resource utilization.

## Figures and Tables

**Table 1:** The list of obtained samples from dandelion, milk thistle, and chamomile seeds.

No.	Sample Abbreviation	Plant Material/Method of Extraction/Solvent
1.	D_SFE/scCO_2_	Dandelion/Supercritical fluid extraction/sc-CO_2_
2.	D_SX/Et	Dandelion/Soxhlet extraction/absolute ethanol
3.	D_SX/Et/H_2_O	Dandelion/Soxhlet extraction/50% ethanol
4.	D_US/Et	Dandelion/Ultrasound extraction/absolute ethanol
5.	D_US/Et/H_2_O	Dandelion/Ultrasound extraction/50% ethanol
6.	D^EX^_SX/Et	Dandelion after SFE/Soxhlet extraction/absolute ethanol
7.	D^EX^_SX/Et/H_2_O	Dandelion after SFE/Soxhlet extraction/50% ethanol
8.	MT_SFE/scCO_2_	Milk thistle/Supercritical fluid extraction/sc-CO_2_
9.	MT_SX/Et	Milk thistle/Soxhlet extraction/absolute ethanol
10.	MT_SX/Et/H_2_O	Milk thistle/Soxhlet extraction/50% ethanol
11.	MT_US/Et	Milk thistle/Ultrasound extraction/absolute ethanol
12.	MT_US/Et/H_2_O	Milk thistle/Ultrasound extraction/50% ethanol
13.	MT^EX^_SX/Et	Milk thistle after SFE/Soxhlet extraction/absolute ethanol
14.	MT^EX^_SX/Et/H_2_O	Milk thistle after SFE/Soxhlet extraction/50% ethanol
15.	C_SFE/scCO_2_	Chamomile/Supercritical fluid extraction/sc-CO_2_
16.	C_SX/Et	Chamomile/Soxhlet extraction/absolute ethanol
17.	C_SX/Et/H_2_O	Chamomile/Soxhlet extraction/50% ethanol
18.	C_US/Et	Chamomile/Ultrasound extraction/absolute ethanol
19.	C_US/Et/H_2_O	Chamomile/Ultrasound extraction/50% ethanol
20.	C^EX^_SX/Et	Chamomile after SFE/Soxhlet extraction/absolute ethanol
21.	C^EX^_SX/Et/H_2_O	Chamomile after SFE/Soxhlet extraction/50% ethanol

**Table 2:** Pigment content and antioxidant activity for samples from native and waste dandelion, milk thistle, and chamomile seeds.

Sample	Chlorophyll A (mg/kg)	Chlorophyll B (mg/kg)	Carotenoids (mg/kg)	IC_50_ (mg/mL)
D_SFE/sc-CO_2_	95.86 ± 0.98 ^c^	23.67 ± 0.44 ^d^	31.14 ± 0.24 ^b,c^	51.04 ± 0.11 ^a^
D_SX/Et	96.88 ± 14.58 ^b,c^	74.24 ± 14.01 ^c^	25.61 ± 2.72 ^c^	0.78 ± 0.01 ^c^
D_SX/Et/H_2_O	65.35 ± 1.81 ^c^	119.57 ± 2.34 ^b^	0.10 ± 0.70 ^d^	0.77 ± 0.01 ^c^
D_US/Et	129.38 ± 4.82 ^b^	56.60 ± 3.25 ^c^	32.75 ± 1.28 ^b^	1.23 ± 0.01 ^b^
D_US/Et/H_2_O	21.74 ± 0.49 ^d^	27.70 ± 0.50 ^d^	5.86 ± 0.08 ^d^	0.27 ± 0.06 ^d^
D^EX^_SX/Et	186.73 ± 0.42 ^a^	170.78 ± 1.20 ^a^	94.23 ± 0.39 ^a^	0.29 ± 0.01 ^d^
D^EX^_SX/Et/H_2_O	32.20 ± 1.24 ^d^	51.57 ± 1.63 ^c,d^	4.64 ± 0.31 ^d^	0.52 ± 0.04 ^c,d^
MT_SFE/sc-CO_2_	22.02 ± 0.68 ^c^	23.80 ± 0.15 ^c^	8.15 ± 0.11 ^c,d^	30.91 ± 0.88 ^a^
MT_SX/Et	72.78 ± 7.17 ^a^	79.37 ± 7.48 ^a^	18.96 ± 2.82 ^b^	1.08 ± 0.04 ^c^
MT_SX/Et/H_2_O	17.16 ± 2.00 ^c^	27.22 ± 2.00 ^c^	3.52 ± 0.50 ^c,d^	0.40 ± 0.03 ^c^
MT_US/Et	89.10 ± 2.76 ^a^	53.27 ± 2.61 ^b^	26.41 ± 0.64 ^a^	3.30 ± 0.11 ^b^
MT_US/Et/H_2_O	17.98 ± 0.40 ^c^	26.17 ± 0.24 ^c^	2.69 ± 0.21 ^d^	0.67 ± 0.07 ^c^
MT^EX^_SX/Et	40.29 ± 1.39 ^b^	51.50 ± 1.60 ^b^	27.02 ± 1.08 ^a^	0.45 ± 0.01 ^c^
MT^EX^_SX/Et/H_2_O	24.06 ± 2.39 ^b,c^	40.84 ± 2.51 ^b,c^	9.58 ± 0.66 ^c^	0.30 ± 0.01 ^c^
C_SFE/sc-CO_2_	146.28 ± 2.66 ^d^	31.90 ± 1.13 ^d^	76.00 ± 0.62 ^c^	17.64 ± 0.47 ^a^
C_SX/Et	550.14 ± 15.01 ^b^	201.91 ± 13.24 ^b,c^	120.11 ± 3.08 ^b^	0.47 ± 0.01 ^d^
C_SX/Et/H_2_O	270.67 ± 2.37 ^c^	286.32 ± 37.88 ^b^	23.26 ± 7.92 ^d,e^	0.46 ± 0.02 ^d^
C_US/Et	573.21 ± 11.32 ^b^	239.32 ± 7.10 ^b^	168.93 ± 2.50 ^a^	2.84 ± 0.10 ^b^
C_US/Et/H_2_O	37.27 ± 1.34 ^e^	29.43 ± 1.23 ^d^	8.14 ± 0.40 ^e^	2.36 ± 0.45 ^b,c^
C^EX^_SX/Et	805.45 ± 3.24 ^a^	373.27 ± 0.56 ^a^	178.55 ± 0.70 ^a^	0.39 ± 0.01 ^d^
C^EX^_SX/Et/H_2_O	229.62 ± 3.54 ^c^	144.71 ± 2.40 ^c^	37.92 ± 0.96 ^d^	0.87 ± 0.05 ^c,d^

IC_50_—concentration of oil or extract required for the 50% decrease in absorbance of the DPPH control solution. Different letters (a–e) in superscript represent statistically significant differences (*p* < 0.05) (comparisons were made column-wise for each plant material individually).

**Table 3:** Cytotoxicity and anticancer selectivity of extracts from dandelion, milk thistle, and chamomile seeds.

Sample	VERO CC_50_ (µg/mL)	FaDu		HeLa		RKO	
		CC_50_ (µg/mL)	SI	CC_50_ (µg/mL)	SI	CC_50_ (µg/mL)	SI
D_SX/Et/H_2_O	794.65 ± 41.08	347.40 ± 45.25	2.29	645.80 ± 28.14	1.23	974.90 ± 97.72	0.82
D^EX^_SX/Et/H_2_O	578.15 ± 56.07	274.60 ± 26.87	2.11	360.20 ± 30.12	1.61	633.95 ± 57.35	0.91
MT_SX/Et/H_2_O	480.55 ± 6.29	190.05 ± 17.47	2.53	254.50 ± 31.54	1.89	270.55 ± 13.65	1.78
MT^EX^_SX/Et/H_2_O	493.60 ± 8.06	182.90 ± 6.65	2.70	230.85 ± 28.50	2.14	264.15 ± 12.23	1.87
C_SX/Et/H_2_O	200.55 ± 22.84	363.90 ± 40.16	0.55	678.15 ± 53.81	0.30	627.95 ± 45.89	0.32
C^EX^_SX/Et/H_2_O	264.15 ± 16.19	293.30 ± 34.22	0.90	345.05 ± 8.84	0.77	777.00 ± 28.14	0.34

VERO—non-cancerous monkey kidney cells; FaDu—human hypopharyngeal squamous cell carcinoma cells; HeLa—human cervical adenocarcinoma cells; RKO—human colon cancer cells; CC_50_—concentration resulting in a 50% reduction in cell viability; SI—Selectivity Index (CC_50_ VERO/CC_50_ cancer cells).

## Data Availability

The original contributions presented in the study are included in the article/Supplementary Materials, further inquiries can be directed to the corresponding author.

## Supplementary Materials

The following supporting information can be downloaded at https://www.mdpi.com/article/10.3390/foods13233907/s1: Table S1: Extraction yield data from replicate experiments for native and waste seeds of dandelion (D), milk thistle (MT), and chamomile (C) obtained by SFE, SXE, and USE using sc-CO_2_, absolute ethanol, or aqueous ethanol as solvents; Table S2: *P*-values for extraction yield data from Table S1; Table S3: Total phenolic content data from replicate experiments for native and waste seeds of dandelion (D), milk thistle (MT), and chamomile (C) obtained by SFE, SXE, and USE using sc-CO_2_, absolute ethanol, or aqueous ethanol as solvents; Table S4: *p*-values for total phenolic content data from Table S3; Table S5: Comparison of the total phenolic content data recorded in the current study with the literature reports for the extracts from dandelion (D), milk thistle (MT), and chamomile (C); Table S6: Total flavonoid content data from replicate experiments for native and waste seeds of dandelion (D), milk thistle (MT), and chamomile (C) obtained by SFE, SXE, and USE using sc-CO_2_, absolute ethanol, or aqueous ethanol as solvents; Table S7: *p*-values for total flavonoid content data from Table S6; Table S8: Comparison of the total flavonoid content data recorded in the current study with the literature reports for the extracts from dandelion (D), milk thistle (MT), and chamomile (C); Table S9: Chlorophyll A content data from replicate experiments for native and waste seeds of dandelion (D), milk thistle (MT), and chamomile (C) obtained by SFE, SXE, and USE using sc-CO_2_, absolute ethanol, or aqueous ethanol as solvents; Table S10: *p*-values for chlorophyll A content data from Table S9; Table S11: DPPH radical scavenging activity expresses as IC50 (concentration of oil or extract required for the 50% decrease in absorbance of the DPPH control solution) data from replicate experiments for native and waste seeds of dandelion (D), milk thistle (MT), and chamomile (C) obtained by SFE, SXE, and USE using sc-CO_2_, absolute ethanol, or aqueous ethanol as solvents; Table S12: *p*-values for IC_50_ data from Table S11; Table S13: The coefficient of variation (CV) within the cytotoxicity results.
