# Supplementary material for: Optimizing Green Extraction Methods for Maximizing the Biological Potential of Dandelion, Milk Thistle, and Chamomile Seed Extracts
Source: Foods. 2024 Dec 3;13(23):3907. doi: 10.3390/foods13233907 (PMC11640657; doi:10.3390/foods13233907)
Supplement: Supplementary file 1 [file foods-13-03907-s001.zip › foods-3326313-supplementary.pdf]

foods

Supplementary Material

Optimizing Green Extraction Methods for Maximizing the Biological Potential of Dandelion, Milk Thistle, and Chamomile Seed Extracts

Stoja Milovanovic [1,*], Katarzyna Tyśkiewicz [2], Marcin Konkol [2], Agnieszka Grzegorczyk [3], Kinga Salwa [4] and Łukasz Świątek [4]

[1] Faculty of Technology and Metallurgy, University of Belgrade, Karnegijeva 4, 11120 Belgrade, Serbia
[2] Łukasiewicz Research Network—New Chemical Syntheses Institute, Al. Tysiąclecia Państwa Polskiego 13a, 24-110 Puławy, Poland; katarzyna.tyskiewicz@ins.lukasiewicz.gov.pl (K.T.); marcin.konkol@ins.lukasiewicz.gov.pl (M.K.)
[3] Chair and Department of Pharmaceutical Microbiology, Medical University of Lublin, Chodźki 1, 20-093 Lublin, Poland; agnieszka.grzegorczyk@umlub.pl
[4] Department of Virology with Viral Diagnostics Laboratory, Medical University of Lublin, Chodźki 1, 220-093 Lublin, Poland; kinga.salwa@umlub.pl (K.S.); lukasz.swiatek@umlub.pl (Ł.Ś.)
* Correspondence: smilovanovic@tmf.bg.ac.rs; Tel.: +381-113303795

foods

MDPI

Table S1. Extraction yield data from replicate experiments for native and waste seeds of dandelion (D), milk thistle (MT), and chamomile (C) obtained by SFE, SXE, and USE using sc-CO_2, absolute ethanol, or aqueous ethanol as solvents

	Sample	Ymax (%)	Ymin (%)	Mean	Standard deviation
A	D_SFE/sc-CO_2	28.29	24.05	26.17	2.12
B	D_SX/Et	34.00	31.69	32.85	1.16
C	D_SX/Et/H_2O	32.05	28.27	30.16	1.89
D	D_US/Et	25.73	23.60	24.66	1.06
E	D_US/Et/H_2O	15.00	12.95	13.98	1.03
F	D^{EX}_SX/Et	9.50	7.80	8.65	0.85
G	D^{EX}_SX/Et/H_2O	16.66	14.40	15.53	1.13
A	MT_SFE/sc-CO_2	28.71	28.12	28.42	0.30
B	MT_SX/Et	30.90	28.34	29.62	1.28
C	MT_SX/Et/H_2O	10.11	7.61	8.86	1.25
D	MT_US/Et	22.42	21.14	21.78	0.64
E	MT_US/Et/H_2O	13.61	11.39	12.50	1.11
F	MT^{EX}_SX/Et	15.07	13.00	14.03	1.03
G	MT^{EX}_SX/Et/H_2O	8.84	6.84	7.84	1.00
A	C_SFE/sc-CO_2	10.15	7.15	8.65	1.50
B	C_SX/Et	8.40	7.39	7.90	0.51
C	C_SX/Et/H_2O	15.79	13.72	14.76	1.04
D	C_US/Et	5.56	5.36	5.46	0.10
E	C_US/Et/H_2O	14.80	11.80	13.30	1.50
F	C^{EX}_SX/Et	4.95	4.10	4.52	0.42
G	C^{EX}_SX/Et/H_2O	11.40	10.50	10.95	0.45

Table S2. *P*-values for extraction yield data from Table S1

Dandelion			Milk thistle			Chamomile		
treatments pair	Tukey HSD *p*-value	Tukey HSD inferfence	treatments pair	Tukey HSD *p*-value	Tukey HSD inferfence	treatments pair	Tukey HSD *p*-value	Tukey HSD inferfence
A vs B	0.0991325	insignificant	A vs B	0.8999947	insignificant	A vs B	0.8999947	insignificant
A vs C	0.4742483	insignificant	A vs C	0.0010053	** p<0.01	A vs C	0.0247798	* p<0.05
A vs D	0.8999947	insignificant	A vs D	0.0218961	* p<0.05	A vs D	0.3205326	insignificant
A vs E	0.0047492	** p<0.01	A vs E	0.0010053	** p<0.01	A vs E	0.0878777	insignificant
A vs F	0.0010053	** p<0.01	A vs F	0.0010053	** p<0.01	A vs F	0.1410121	insignificant
A vs G	0.0103116	* p<0.05	A vs G	0.0010053	** p<0.01	A vs G	0.6162229	insignificant
B vs C	0.7896741	insignificant	B vs C	0.0010053	** p<0.01	B vs C	0.0134245	* p<0.05
B vs D	0.0402164	* p<0.05	B vs D	0.0089198	** p<0.01	B vs D	0.5677688	insignificant
B vs E	0.0010053	** p<0.01	B vs E	0.0010053	** p<0.01	B vs E	0.0450149	* p<0.05
B vs F	0.0010053	** p<0.01	B vs F	0.0010053	** p<0.01	B vs F	0.2752650	insignificant
B vs G	0.0010053	** p<0.01	B vs G	0.0010053	** p<0.01	B vs G	0.3589965	insignificant

C vs D	0.2030376	insignificant	C vs D	0.0010053	** p<0.01	C vs D
C vs E	0.0010053	** p<0.01	C vs E	0.2611627	insignificant	C vs E
C vs F	0.0010053	** p<0.01	C vs F	0.0717185	insignificant	C vs F
C vs G	0.0015951	** p<0.01	C vs G	0.8999947	insignificant	C vs G
D vs E	0.0100476	* p<0.05	D vs E	0.0033712	** p<0.01	D vs E
D vs F	0.0010053	** p<0.01	D vs F	0.0095468	** p<0.01	D vs F
D vs G	0.0232575	* p<0.05	D vs G	0.0010053	** p<0.01	D vs G
E vs F	0.2247870	insignificant	E vs F	0.8999947	insignificant	E vs F
E vs G	0.8999947	insignificant	E vs G	0.1109207	insignificant	E vs G
F vs G	0.0874980	insignificant	F vs G	0.0309882	* p<0.05	F vs G

Values are significantly different for p<0.05 (comparisons were made for each plant material individually)

foods

MDPI

Table S3. Total phenolic content data from replicate experiments for native and waste seeds of dandelion (D), milk thistle (MT), and chamomile (C) obtained by SFE, SXE, and USE using sc-CO_2, absolute ethanol, or aqueous ethanol as solvents

	Sample	TPCmax (%)	TPCmin (%)	Mean	Standard deviation
A	D_SFE/sc-CO_2	48.98	39.29	44.14	4.85
B	D_SX/Et	260.40	255.58	257.99	2.41
C	D_SX/Et/H_2O	543.72	536.24	539.98	3.74
D	D_US/Et	167.82	163.80	165.81	2.01
E	D_US/Et/H_2O	563.21	501.62	532.41	30.79
F	D^{EX}_SX/Et	576.04	533.39	554.71	21.33
G	D^{EX}_SX/Et/H_2O	988.19	980.07	984.13	4.06
A	MT_SFE/sc-CO_2	40.05	35.68	37.87	2.19
B	MT_SX/Et	676.01	666.32	671.17	4.85
C	MT_SX/Et/H_2O	1803.04	1680.64	1741.84	61.20
D	MT_US/Et	381.55	374.10	377.83	3.73
E	MT_US/Et/H_2O	939.57	916.68	928.13	11.45
F	MT^{EX}_SX/Et	2010.26	1794.68	1902.47	107.79
G	MT^{EX}_SX/Et/H_2O	2163.23	2088.37	2125.80	37.43
A	C_SFE/sc-CO_2	56.50	38.59	47.55	8.96
B	C_SX/Et	382.27	354.55	368.41	13.86
C	C_SX/Et/H_2O	483.20	439.64	461.42	21.78
D	C_US/Et	120.12	119.15	119.63	0.49
E	C_US/Et/H_2O	195.16	176.17	185.66	9.49
F	C^{EX}_SX/Et	471.48	445.02	458.25	13.23
G	C^{EX}_SX/Et/H_2O	823.36	784.44	803.90	19.46

foods

MDPI

Table S4. *P*-values for total phenolic content data from Table S3

Dandelion			Milk thistle			Chamomile		
treatments pair	Tukey HSD *p*-value	Tukey HSD inferfence	treatments pair	Tukey HSD *p*-value	Tukey HSD inferfence	treatments pair	Tukey HSD *p*-value	Tukey HSD inferfence
A vs B	0.0010053	** $p<0.01$	A vs B	0.0010053	** $p<0.01$	A vs B	0.0010053	** $p<0.01$
A vs C	0.0010053	** $p<0.01$	A vs C	0.0010053	** $p<0.01$	A vs C	0.0010053	** $p<0.01$
A vs D	0.0060079	** $p<0.01$	A vs D	0.0176952	* $p<0.05$	A vs D	0.0754530	insignificant
A vs E	0.0010053	** $p<0.01$	A vs E	0.0010053	** $p<0.01$	A vs E	0.0024326	** $p<0.01$
A vs F	0.0010053	** $p<0.01$	A vs F	0.0010053	** $p<0.01$	A vs F	0.0010053	** $p<0.01$
A vs G	0.0010053	** $p<0.01$	A vs G	0.0010053	** $p<0.01$	A vs G	0.0010053	** $p<0.01$
B vs C	0.0010053	** $p<0.01$	B vs C	0.0010053	** $p<0.01$	B vs C	0.0225225	* $p<0.05$
B vs D	0.0270038	* $p<0.05$	B vs D	0.0373342	* $p<0.05$	B vs D	0.0010053	** $p<0.01$
B vs E	0.0010053	** $p<0.01$	B vs E	0.0688207	insignificant	B vs E	0.0010053	** $p<0.01$
B vs F	0.0010053	** $p<0.01$	B vs F	0.0010053	** $p<0.01$	B vs F	0.0268776	* $p<0.05$
B vs G	0.0010053	** $p<0.01$	B vs G	0.0010053	** $p<0.01$	B vs G	0.0010053	** $p<0.01$

C vs D	0.0010053	** *p*<0.01	C vs D	0.0010053	** *p*<0.01	C vs D	0.0010053	** *p*<0.01
C vs E	0.8999947	insignificant	C vs E	0.0010053	** *p*<0.01	C vs E	0.0010053	** *p*<0.01
C vs F	0.8999947	insignificant	C vs F	0.3521726	insignificant	C vs F	0.8999947	insignificant
C vs G	0.0010053	** *p*<0.01	C vs G	0.0091381	** *p*<0.01	C vs G	0.0010053	** *p*<0.01
D vs E	0.0010053	** *p*<0.01	D vs E	0.0010758	** *p*<0.01	D vs E	0.1086050	insignificant
D vs F	0.0010053	** *p*<0.01	D vs F	0.0010053	** *p*<0.01	D vs F	0.0010053	** *p*<0.01
D vs G	0.0010053	** *p*<0.01	D vs G	0.0010053	** *p*<0.01	D vs G	0.0010053	** *p*<0.01
E vs F	0.8999947	insignificant	E vs F	0.0010053	** *p*<0.01	E vs F	0.0010053	** *p*<0.01
E vs G	0.0010053	** *p*<0.01	E vs G	0.0010053	** *p*<0.01	E vs G	0.0010053	** *p*<0.01
F vs G	0.0010053	** *p*<0.01	F vs G	0.1228344	insignificant	F vs G	0.0010053	** *p*<0.01

Values are significantly different for *p*<0.05 (comparisons were made for each plant material individually)

foods

MDPI

Table S5. Comparison of the total phenolic content data recorded in the current study with the literature reports for the extracts from dandelion (D), milk thistle (MT), and chamomile (C)

Current study		Literature			
**Sample**	**TPC (mg GAE/g)**	**Plant material**	**Solvent**	**TPC (mg GAE/g)**	**Reference**
D_SFE/sc-CO_2	44.14 ± 4.85	D seeds	sc-CO_2	5.5–12.1	[22]
D_SX/Et	257.99 ± 2.41	D petals	MeOH/H_2O	253.1	[7]
D_US/Et	165.81 ± 2.01	D seeds	C_6H_{14}	24.1	[16]
D_SX/Et/H_2O	539.98 ± 3.74	D leaf, flower	H_2O	5.3–15.5	[34]
D_US/Et/H_2O	532.41 ± 30.79	D seeds	EtOH	180	[35]
D^{EX}_SX/Et	554.71 ± 21.33	D seeds	MeOH	188	[36]
D^{EX}_SX/Et/H_2O	984.13 ± 4.06	D stems	EtOH/H_2O	41.2	[37]
MT_SFE/sc-CO_2	37.87 ± 2.19	MT seeds	sc-CO_2	9.2–14.2	[21]
MT_SX/Et	671.17 ± 4.85	MT seeds	CO_2/EtOH/H_2O	237–758	[38]
MT_US/Et	377.83 ± 3.73	MT seeds	H_2O	164.8–238.3	[14]
MT_SX/Et/H_2O	1741.84 ± 61.20	MT seeds	C_6H_{14}	1.5	[39]
MT_US/Et/H_2O	928.13 ± 11.45	MT achenes	EtOH	3.5	[40]
MT^{EX}_SX/Et	1902.47 ± 107.79	defatted MT seeds	EtOH	0.6	[41]
MT^{EX}_SX/Et/H_2O	2125.80 ± 37.43	defatted MT seeds	MeOH	30	[42]
C_SFE/sc-CO_2	47.55 ± 8.96	C seeds	sc-CO_2/EtOH	20.3–31.5	[23]
C_SX/Et	368.41 ± 13.86	C flowers	H_2O, C_3H_6O	19.6–143.2	[43]
C_US/Et	119.63 ± 0.49	C seeds	H_2O	16.4–22.4	[31]
C_SX/Et/H_2O	461.42 ± 21.78	C herb	sc-CO_2	2.3	[44]
C_US/Et/H_2O	185.66 ± 9.49	C flowers	EtOH	21.4	[45]
C^{EX}_SX/Et	458.25 ± 13.23	C flowers	EtOH	41.1–100.5	[46]
C^{EX}_SX/Et/H_2O	803.90 ± 19.46	C flowers	MeOH/H_2O	35.5–46.2	[47]

foods MDPI

Table S6. Total flavonoid content data from replicate experiments for native and waste seeds of dandelion (D), milk thistle (MT), and chamomile (C) obtained by SFE, SXE, and USE using sc-CO_2, absolute ethanol, or aqueous ethanol as solvents

	Sample	TFCmax (%)	TFCmin (%)	Mean	Standard deviation
A	D_SFE/sc-CO_2	0.62	0.60	0.61	0.01
B	D_SX/Et	3.54	3.38	3.46	0.08
C	D_SX/Et/H_2O	3.48	3.47	3.48	0.01
D	D_US/Et	3.50	2.50	3.00	0.50
E	D_US/Et/H_2O	4.00	2.80	3.40	0.60
F	D^{EX}_SX/Et	12.05	11.78	11.92	0.13
G	D^{EX}_SX/Et/H_2O	6.75	6.43	6.59	0.16
A	MT_SFE/sc-CO_2	0.26	0.24	0.25	0.01
B	MT_SX/Et	7.35	7.20	7.27	0.08
C	MT_SX/Et/H_2O	19.37	19.16	19.27	0.10
D	MT_US/Et	3.47	3.29	3.38	0.09
E	MT_US/Et/H_2O	9.74	9.24	9.49	0.25
F	MT^{EX}_SX/Et	19.07	18.81	18.94	0.13
G	MT^{EX}_SX/Et/H_2O	26.09	25.60	25.85	0.24
A	C_SFE/sc-CO_2	1.03	1.00	1.01	0.01
B	C_SX/Et	5.32	5.17	5.24	0.07
C	C_SX/Et/H_2O	4.90	3.70	4.30	0.60
D	C_US/Et	3.84	3.79	3.81	0.03
E	C_US/Et/H_2O	1.60	1.52	1.56	0.04
F	C^{EX}_SX/Et	3.26	3.24	3.25	0.01
G	C^{EX}_SX/Et/H_2O	3.39	3.13	3.26	0.13

Table S7. *P*-values for total flavonoid content data from Table S6

Dandelion			Milk thistle			Chamomile		
treatments pair	Tukey HSD *p*-value	Tukey HSD inferfence	treatments pair	Tukey HSD *p*-value	Tukey HSD inferfence	treatments pair	Tukey HSD *p*-value	Tukey HSD inferfence
A vs B	0.0033720	** p<0.01	A vs B	0.0010053	** p<0.01	A vs B	0.0010053	** p<0.01
A vs C	0.0032678	** p<0.01	A vs C	0.0010053	** p<0.01	A vs C	0.0010053	** p<0.01
A vs D	0.0092986	** p<0.01	A vs D	0.0010053	** p<0.01	A vs D	0.0010053	** p<0.01
A vs E	0.0038258	** p<0.01	A vs E	0.0010053	** p<0.01	A vs E	0.6556080	insignificant
A vs F	0.0010053	** p<0.01	A vs F	0.0010053	** p<0.01	A vs F	0.0028783	** p<0.01
A vs G	0.0010053	** p<0.01	A vs G	0.0010053	** p<0.01	A vs G	0.0028026	** p<0.01
B vs C	0.8999947	insignificant	B vs C	0.0010053	** p<0.01	B vs C	0.1891015	insignificant
B vs D	0.8999947	insignificant	B vs D	0.0010053	** p<0.01	B vs D	0.0335148	* p<0.05
B vs E	0.8999947	insignificant	B vs E	0.0010053	** p<0.01	B vs E	0.0010053	** p<0.01
B vs F	0.0010053	** p<0.01	B vs F	0.0010053	** p<0.01	B vs F	0.0056096	** p<0.01
B vs G	0.0019163	** p<0.01	B vs G	0.0010053	** p<0.01	B vs G	0.0057748	** p<0.01

C vs D	0.8999947	insignificant	C vs D	0.0010053	** $p<0.01$	C vs D	0.7421540	insignificant
C vs E	0.8999947	insignificant	C vs E	0.0010053	** $p<0.01$	C vs E	0.0010053	** $p<0.01$
C vs F	0.0010053	** $p<0.01$	C vs F	0.7239946	insignificant	C vs F	0.1294207	insignificant
C vs G	0.0019755	** $p<0.01$	C vs G	0.0010053	** $p<0.01$	C vs G	0.1342122	insignificant
D vs E	0.8999947	insignificant	D vs E	0.0010053	** $p<0.01$	D vs E	0.0027289	** $p<0.01$
D vs F	0.0010053	** $p<0.01$	D vs F	0.0010053	** $p<0.01$	D vs F	0.6267592	insignificant
D vs G	0.0010053	** $p<0.01$	D vs G	0.0010053	** $p<0.01$	D vs G	0.6411824	insignificant
E vs F	0.0010053	** $p<0.01$	E vs F	0.0010053	** $p<0.01$	E vs F	0.0141686	* $p<0.05$
E vs G	0.0017060	** $p<0.01$	E vs G	0.0010053	** $p<0.01$	E vs G	0.0137245	* $p<0.05$
F vs G	0.0010053	** $p<0.01$	F vs G	0.0010053	** $p<0.01$	F vs G	0.8999947	insignificant

Values are significantly different for $p<0.05$ (comparisons were made for each plant material individually)

foods

MDPI

Table S8. Comparison of the total flavonoid content data recorded in the current study with the literature reports for the extracts from dandelion (D), milk thistle (MT), and chamomile (C)

Current study		Literature			
**Sample**	**TFC (mg QE/g)**	**Plant material**	**Solvent**	**TFC (mg QE/g)**	**Reference**
D_SFE/sc-CO_2	0.61 ± 0.01	D seeds	sc-CO_2	0.2–0.6	[22]
D_SX/Et	3.46 ± 0.08	D seeds	C_6H_{14}	1.0	[16]
D_US/Et	3.00 ± 0.50	D leaf, flower	EtOH/H_2O	5.0–2.3	[49]
D_SX/Et/H_2O	3.48 ± 0.01	D leaves	MeOH	0.7	[50]
D_US/Et/H_2O	3.40 ± 0.60	D leaves	MeOH	3.6*	[51]
D^{EX}_SX/Et	11.92 ± 0.13	D roots	H_2O	2.0–2.2	[52]
D^{EX}_SX/Et/H_2O	6.59 ± 0.16				
MT_SFE/sc-CO_2	0.25 ± 0.01	MT seeds	sc-CO_2	0.10–0.16	[21]
MT_SX/Et	7.27 ± 0.08	MT seeds	petroleum ether	0.16–0.21	[53]
MT_US/Et	3.38 ± 0.09	defatted MT seeds	EtOH	0.04	[41]
MT_SX/Et/H_2O	19.27 ± 0.10	MT seeds	H_2O	3.3	[54]
MT_US/Et/H_2O	9.49 ± 0.25	defatted MT seeds	EtOH/H_2O	10.5	[55]
MT^{EX}_SX/Et	18.94 ± 0.13	MT seeds	-	4.4·10-3	[56]
MT^{EX}_SX/Et/H_2O	25.85 ± 0.24	MT callus	MeOH	3–10	[57]
C_SFE/sc-CO_2	1.01 ± 0.01	C seeds	sc-CO_2/EtOH	0.8–4.2	[23]
C_SX/Et	5.24 ± 0.07	C seeds	H_2O	1.1–1.8	[31]
C_US/Et	3.81 ± 0.03	C flowers	MeOH/H_2O	23.0–30.5*	[47]
C_SX/Et/H_2O	4.30 ± 0.60	C root, stem	H_2O	2.1–4.3	[59]
C_US/Et/H_2O	1.56 ± 0.04	C flowers	MeOH	4.7	[60]
C^{EX}_SX/Et	3.25 ± 0.01	C flowers	H_2O	0.7	[61]
C^{EX}_SX/Et/H_2O	3.26 ± 0.13				

*results for this sample from literature were expressed as milligrams of catechin equivalents per g of extracts

foods

MDPI

Table S9. Chlorophyll A content data from replicate experiments for native and waste seeds of dandelion (D), milk thistle (MT), and chamomile (C) obtained by SFE, SXE, and USE using sc-CO_2, absolute ethanol, or aqueous ethanol as solvents

	**Sample**	Chlorophyll A **max (mg/kg)**	Chlorophyll A **min (mg/kg)**	**Mean**	**Standard deviation**
A	D_SFE/sc-CO_2	96.84	94.87	95.86	0.98
B	D_SX/Et	111.45	82.30	96.88	14.58
C	D_SX/Et/H_2O	67.17	63.54	65.35	1.81
D	D_US/Et	134.19	124.56	129.38	4.82
E	D_US/Et/H_2O	22.23	21.24	21.74	0.49
F	D^{EX}_SX/Et	187.14	186.31	186.73	0.42
G	D^{EX}_SX/Et/H_2O	33.44	30.96	32.20	1.24
A	MT_SFE/sc-CO_2	22.70	21.34	22.02	0.68
B	MT_SX/Et	79.96	65.61	72.78	7.17
C	MT_SX/Et/H_2O	19.16	15.16	17.16	2.00
D	MT_US/Et	91.86	86.35	89.10	2.76
E	MT_US/Et/H_2O	18.37	17.58	17.98	0.40
F	MT^{EX}_SX/Et	41.68	38.90	40.29	1.39
G	MT^{EX}_SX/Et/H_2O	26.45	21.68	24.06	2.39
A	C_SFE/sc-CO_2	148.95	143.62	146.28	2.66
B	C_SX/Et	565.15	535.13	550.14	15.01
C	C_SX/Et/H_2O	273.04	268.29	270.67	2.37
D	C_US/Et	584.53	561.88	573.21	11.32
E	C_US/Et/H_2O	38.62	35.93	37.27	1.34
F	C^{EX}_SX/Et	808.70	802.21	805.45	3.24
G	C^{EX}_SX/Et/H_2O	233.17	226.08	229.62	3.54

foods

MDPI

Table S10. *P*-values for chlorophyll A content data from Table S9

Dandelion			Milk thistle			Chamomile		
treatments pair	Tukey HSD *p*-value	Tukey HSD inferfence	treatments pair	Tukey HSD *p*-value	Tukey HSD inferfence	treatments pair	Tukey HSD *p*-value	Tukey HSD inferfence
A vs B	0.8999947	insignificant	A vs B	0.0010053	** $p<0.01$	A vs B	0.0010053	** $p<0.01$
A vs C	0.0708545	insignificant	A vs C	0.8999947	insignificant	A vs C	0.0010053	** $p<0.01$
A vs D	0.0461900	* $p<0.05$	A vs D	0.0010053	** $p<0.01$	A vs D	0.0010053	** $p<0.01$
A vs E	0.0010053	** $p<0.01$	A vs E	0.8999947	insignificant	A vs E	0.0010053	** $p<0.01$
A vs F	0.0010053	** $p<0.01$	A vs F	0.0453867	* $p<0.05$	A vs F	0.0010053	** $p<0.01$
A vs G	0.0013210	** $p<0.01$	A vs G	0.8999947	insignificant	A vs G	0.0011011	** $p<0.01$
B vs C	0.0612606	insignificant	B vs C	0.0010053	** $p<0.01$	B vs C	0.0010053	** $p<0.01$
B vs D	0.0533180	insignificant	B vs D	0.0755276	insignificant	B vs D	0.4047653	insignificant
B vs E	0.0010053	** $p<0.01$	B vs E	0.0010053	** $p<0.01$	B vs E	0.0010053	** $p<0.01$
B vs F	0.0010053	** $p<0.01$	B vs F	0.0019277	** $p<0.01$	B vs F	0.0010053	** $p<0.01$
B vs G	0.0011946	** $p<0.01$	B vs G	0.0010053	** $p<0.01$	B vs G	0.0010053	** $p<0.01$

C vs D	0.0012697	** p<0.01	C vs D	0.0010053	** p<0.01	C vs D	0.0010053	** p<0.01
C vs E	0.0120764	* p<0.05	C vs E	0.8999947	insignificant	C vs E	0.0010053	** p<0.01
C vs F	0.0010053	** p<0.01	C vs F	0.0137348	* p<0.05	C vs F	0.0010053	** p<0.01
C vs G	0.0486206	* p<0.05	C vs G	0.7098215	insignificant	C vs G	0.0551010	insignificant
D vs E	0.0010053	** p<0.01	D vs E	0.0010053	** p<0.01	D vs E	0.0010053	** p<0.01
D vs F	0.0024959	** p<0.01	D vs F	0.0010053	** p<0.01	D vs F	0.0010053	** p<0.01
D vs G	0.0010053	** p<0.01	D vs G	0.0010053	** p<0.01	D vs G	0.0010053	** p<0.01
E vs F	0.0010053	** p<0.01	E vs F	0.0166430	* p<0.05	E vs F	0.0010053	** p<0.01
E vs G	0.8393821	insignificant	E vs G	0.7962093	insignificant	E vs G	0.0010053	** p<0.01
F vs G	0.0010053	** p<0.01	F vs G	0.0774501	insignificant	F vs G	0.0010053	** p<0.01

Values are significantly different for p<0.05 (comparisons were made for each plant material individually)

Table S11. DPPH radical scavenging activity expresses as IC_{50} (concentration of oil or extract required for the 50% decrease in absorbance of the DPPH control solution) data from replicate experiments for native and waste seeds of dandelion (D), milk thistle (MT), and chamomile (C) obtained by SFE, SXE, and USE using sc-CO_2, absolute ethanol, or aqueous ethanol as solvents

	**Sample**	IC_{50} **max (mg/mL)**	IC_{50} **min (mg/mL)**	**Mean**	**Standard deviation**
A	D_SFE/sc-CO_2	51.15	50.93	51.04	0.11
B	D_SX/Et	0.79	0.77	0.78	0.01
C	D_SX/Et/H_2O	0.78	0.76	0.77	0.01
D	D_US/Et	1.24	1.22	1.23	0.01
E	D_US/Et/H_2O	0.33	0.21	0.27	0.06
F	D^{EX}_SX/Et	0.30	0.28	0.29	0.01
G	D^{EX}_SX/Et/H_2O	0.56	0.48	0.52	0.04
A	MT_SFE/sc-CO_2	31.79	30.03	30.91	0.88
B	MT_SX/Et	1.12	1.039	1.08	0.04
C	MT_SX/Et/H_2O	0.43	0.37	0.40	0.03
D	MT_US/Et	3.41	3.19	3.30	0.11
E	MT_US/Et/H_2O	0.74	0.60	0.67	0.07
F	MT^{EX}_SX/Et	0.46	0.44	0.45	0.01
G	MT^{EX}_SX/Et/H_2O	0.31	0.29	0.30	0.01
A	C_SFE/sc-CO_2	18.11	17.17	17.64	0.47
B	C_SX/Et	0.48	0.46	0.47	0.01
C	C_SX/Et/H_2O	0.48	0.44	0.46	0.02
D	C_US/Et	2.94	2.74	2.84	0.10
E	C_US/Et/H_2O	2.81	1.91	2.36	0.45
F	C^{EX}_SX/Et	0.40	0.38	0.39	0.01
G	C^{EX}_SX/Et/H_2O	1.37	0.37	0.87	0.50

Table S12. *P*-values for IC_{50} data from Table S11

Dandelion			Milk thistle			Chamomile		
treatments pair	Tukey HSD *p*-value	Tukey HSD inferfence	treatments pair	Tukey HSD *p*-value	Tukey HSD inferfence	treatments pair	Tukey HSD *p*-value	Tukey HSD inferfence
A vs B	0.0010053	** $p<0.01$	A vs B	0.0010053	** p<0.01	A vs B	0.0010053	** $p<0.01$
A vs C	0.0010053	** $p<0.01$	A vs C	0.0010053	** $p<0.01$	A vs C	0.0010053	** $p<0.01$
A vs D	0.0010053	** $p<0.01$	A vs D	0.0010053	** $p<0.01$	A vs D	0.0010053	** $p<0.01$
A vs E	0.0010053	** $p<0.01$	A vs E	0.0010053	** $p<0.01$	A vs E	0.0010053	** $p<0.01$
A vs F	0.0010053	** $p<0.01$	A vs F	0.0010053	** $p<0.01$	A vs F	0.0010053	** $p<0.01$
A vs G	0.0010053	** $p<0.01$	A vs G	0.0010053	** $p<0.01$	A vs G	0.0010053	** $p<0.01$
B vs C	0.8999947	insignificant	B vs C	0.7592234	insignificant	B vs C	0.8999947	insignificant
B vs D	0.0041780	** $p<0.01$	B vs D	0.0226279	* $p<0.05$	B vs D	0.0107418	* $p<0.05$
B vs E	0.0019750	** $p<0.01$	B vs E	0.8999947	insignificant	B vs E	0.0349088	* $p<0.05$
B vs F	0.0025159	** $p<0.01$	B vs F	0.8094444	insignificant	B vs F	0.8999947	insignificant
B vs G	0.0719697	insignificant	B vs G	0.6587810	insignificant	B vs G	0.8999947	insignificant

C vs D	0.0036713	** $p<0.01$	C vs D	0.0052311	** $p<0.01$	C vs D	0.0104924	* $p<0.05$
C vs E	0.0022279	** $p<0.01$	C vs E	0.8999947	insignificant	C vs E	0.0340208	* $p<0.05$
C vs F	0.0028484	** $p<0.01$	C vs F	0.8999947	insignificant	C vs F	0.8999947	insignificant
C vs G	0.0851183	insignificant	C vs G	0.8999947	insignificant	C vs G	0.8999947	insignificant
D vs E	0.0010053	** $p<0.01$	D vs E	0.0091193	** $p<0.01$	D vs E	0.8999947	insignificant
D vs F	0.0010053	** $p<0.01$	D vs F	0.0057818	** $p<0.01$	D vs F	0.0089323	** $p<0.01$
D vs G	0.0010053	** $p<0.01$	D vs G	0.0042936	** $p<0.01$	D vs G	0.0284464	* $p<0.05$
E vs F	0.8999947	insignificant	E vs F	0.8999947	insignificant	E vs F	0.0284464	* $p<0.05$
E vs G	0.0851183	insignificant	E vs G	0.8999947	insignificant	E vs G	0.1012609	insignificant
F vs G	0.1193416	insignificant	F vs G	0.8999947	insignificant	F vs G	0.8999947	insignificant

Values are significantly different for $p<0.05$ (comparisons were made for each plant material individually)

Table S13. The coefficient of variation (CV) within the cytotoxicity results

	Coefficient of variation (CV) [%]			
**Sample**	VERO	FaDu	HeLa	RKO
D_SX/Et/H_2O	5.17	13.03	4.36	10.02
D^{EX}_SX/Et/H_2O	9.70	9.79	8.36	9.05
MT_SX/Et/H_2O	1.31	9.19	12.39	5.04
MT^{EX}_SX/Et/H_2O	1.63	3.63	12.34	4.63
C_SX/Et/H_2O	11.39	11.04	7.93	7.31
C^{EX}_SX/Et/H_20	6.13	11.67	2.56	3.62

7. Lis, B.; Jędrejek, D.; Stochmal, A.; Olas, B. Assessment of Effects of Phenolic Fractions from Leaves and Petals of Dandelion in Selected Components of Hemostasis. *Food Res. Int.* **2018**, *107*, 605–612. https://doi.org/10.1016/j.foodres.2018.03.012.

14. Lukic, I.; Milovanovic, S.; Pantic, M.; Srbljak, I.; Djuric, A.; Tadic, V.; Tyśkiewicz, K. Separation of High-Value Extracts from *Silybum marianum* Seeds: Influence of Extraction Technique and Storage on Composition and Bioactivity. *Lwt* **2022**, *160*, 113319. https://doi.org/10.1016/j.lwt.2022.113319.

16. Milovanovic, S.; Grzegorczyk, A.; Świątek, Ł.; Boguszewska, A.; Kowalski, R.; Tyśkiewicz, K.; Konkol, M. Phenolic, Tocopherol, and Essential Fatty Acid-Rich Extracts from Dandelion Seeds: Chemical Composition and Biological Activity. *Food Bioprod. Process.* **2023**, *142*, 70–81. https://doi.org/10.1016/j.fbp.2023.09.005.

21. Milovanovic, S.; Lukic, I.; Kamiński, P.; Dębczak, A.; Klimkowska, K.; Tyśkiewicz, K.; Konkol, M. Green Manufacturing of High-Value Extracts from Milk Thistle Seeds: Parameters That Affect the Supercritical CO_2 Extraction Process. *J. CO_2 Util.* **2022**, *63*, 102134. https://doi.org/10.1016/j.jcou.2022.102134.

22. Milovanovic, S.; Grzegorczyk, A.; Świątek, Ł.; Dębczak, A.; Tyskiewicz, K.; Konkol, M. Dandelion Seeds as a New and Valuable Source of Bioactive Extracts Obtained Using the Supercritical Fluid Extraction Technique. *Sustain. Chem. Pharm.* **2022**, *29*, 100796. https://doi.org/10.1016/j.scp.2022.100796.

23. Milovanovic, S.; Grzegorczyk, A.; Świątek, Ł.; Grzęda, A.; Dębczak, A. A Novel Strategy for the Separation of Functional Oils from Chamomile Seeds. *Food Bioprocess Technol.* **2023**,*16*, 1806–1821. https://doi.org/10.1007/s11947-023-03038-9.

31. Osman, M.; Taie, H.A.; Helmy, W.; Amer, H. Screening for Antioxidant, Antifungal, and Antitumor Activities of Aqueous Extracts of Chamomile (*Matricaria chamomilla*). *Egypt. Pharm. J.* **2016**, *15*, 55. https://doi.org/10.4103/1687-4315.190402.

34. Hudec, J.; Burdová, M.; Kobida, L.; Komora, L.; Macho, V.; Kogan, G.; Turianica, I.; Kochanová, R.; Ložek, O.; Habán, M.; et al. Antioxidant Capacity Changes and Phenolic Profile of *Echinacea Purpurea*, Nettle (*Urtica Dioica* L.), and Dandelion (*Taraxacum Officinale*) after Application of Polyamine and Phenolic Biosynthesis Regulators. *J. Agric. Food Chem.* **2007**, *55*, 5689–5696. https://doi.org/10.1021/jf070777c.

35. Colle, D.; Arantes, L.P.; Rauber, R.; De Mattos, S.E.C.; Rocha, J.B.T. Da; Nogueira, C.W.; Soares, F.A.A. Antioxidant Properties of *Taraxacum Officinale* Fruit Extract Are Involved in the Protective Effect against Cellular Death Induced by Sodium Nitroprusside in Brain of Rats. *Pharm. Biol.* **2012**, *50*, 883–891. https://doi.org/10.3109/13880209.2011.641981.

36. Lis, B.; Jedrejek, D.; Rywaniak, J.; Soluch, A.; Stochmal, A.; Olas, B. Flavonoid Preparations from *Taraxacum Officinale* L. Fruits—A Phytochemical, Antioxidant and Hemostasis Studies. *Molecules* **2020**, *25*, 5402. https://doi.org/10.3390/MOLECULES25225402.

37. Xie, P. jun; Huang, L. xin; Zhang, C. hong; Ding, S. sha; Deng, Y. jun; Wang, X. jie Skin-Care Effects of Dandelion Leaf Extract and Stem Extract: Antioxidant Properties, Tyrosinase Inhibitory and Molecular Docking Simulations. *Ind. Crops Prod.* **2018**, *111*, 238–246. https://doi.org/10.1016/j.indcrop.2017.10.017.

38. Abderrezag, N.; Montenegro, Z.J.S.; Louaer, O.; Meniai, A.H.; Cifuentes, A.; Ibáñez, E.; Mendiola, J.A. One-Step Sustainable Extraction of Silymarin Compounds of Wild Algerian Milk Thistle (*Silybum marianum*) Seeds Using Gas Expanded Liquids. *J. Chromatogr. A* **2022**, *1675*, 463147. https://doi.org/10.1016/j.chroma.2022.463147.

39. Ismaili, S.A.; Marmouzi, I.; Sayah, K.; Harhar, H.; Faouzi, M.E.A.; Gharby, S.; Himmi, B.; Kitane, S.; Belghiti, M.A. El Chemical Analysis and Anti-Oxidation Activities of the Moroccan Milk Thistle. *Moroccan J. Chem*. **2016**, *4*, 695–702. https://doi.org/10.48317/IMIST.PRSM/morjchem-v4i3.4845.

40. Lucini, L.; Kane, D.; Pellizzoni, M.; Ferrari, A.; Trevisi, E.; Ruzickova, G.; Arslan, D. Phenolic Profile and in Vitro Antioxidant Power of Different Milk Thistle [Silybum Marianum (L.) Gaertn.] Cultivars. *Ind. Crops Prod.* **2016**, *83*, 11–16. https://doi.org/10.1016/j.indcrop.2015.12.023.

41. Serçe, A.; Toptanci, B.Ç.; Tanrikut, S.E.; Altas, S.; Kizil, G.; Kizil, S.; Kizil, M. Assessment of the Antioxidant Activity of Silybum Marianum Seed Extract and Its Protective Effect against DNA Oxidation, Protein Damage and Lipid Peroxidation. *Food Technol. Biotechnol*. **2016**, *54*, 455–461. https://doi.org/10.17113/ft b.54.04.16.4323.

42. Aziz, M.; Saeed, F.; Ahmad, N.; Ahmad, A.; Afzaal, M.; Hussain, S.; Mohamed, A.A.; Alamri, M.S.; Anjum, F.M. Biochemical Profile of Milk Thistle (*Silybum Marianum* L.) with Special Reference to Silymarin Content. *Food Sci. Nutr*. **2021**, *9*, 244–250. https://doi.org/10.1002/fsn3.1990.

43. Baranauskienė, R.; Venskutonis, P.R.; Ragažinskienė, O. Valorisation of Roman Chamomile (*Chamaemelum Nobile* L.) Herb by Comprehensive Evaluation of Hydrodistilled Aroma and Residual Non-Volatile Fractions. *Food Res. Int.* **2022**, *160*, 111715. https://doi.org/10.1016/j.foodres.2022.111715.

44. Čižmek, L.; Kralj, M.B.; Čož - rakovac, R.; Mazur, D.; Ul'yanovskii, N.; Likon, M.; Trebše, P. Supercritical Carbon Dioxide Extraction of Four Medicinal Mediterranean Plants: Investigation of Chemical Composition and Antioxidant Activity. *Molecules* **2021**, *26*, 5697. https://doi.org/10.3390/molecules26185697.

45. Al-Dabbagh, B.; Elhaty, I.A.; Elhaw, M.; Murali, C.; Al Mansoori, A.; Awad, B.; Amin, A. Antioxidant and Anticancer Activities of Chamomile (*Matricaria Recutita* L.). *BMC Res. Notes* **2019**, *12*, 1–8. https://doi.org/10.1186/s13104-018-3960-y.

46. Catani, M.V.; Rinaldi, F.; Tullio, V.; Gasperi, V.; Savini, I. Comparative Analysis of Phenolic Composition of Six Commercially Available Chamomile (*Matricaria Chamomilla* l.) Extracts: Potential Biological Implications. *Int. J. Mol. Sci.* **2021**, *22*, 10601. https://doi.org/10.3390/ijms221910601.

47. Wang, Z.; Wu, Z.; Zuo, G.; Lim, S.S.; Yan, H. Defatted Seeds of *Oenothera Biennis* as a Potential Functional Food Ingredient for Diabetes. *Foods* **2021**, *10*, 538. https://doi.org/10.3390/foods10030538.

49. Tsivelika, N.; Irakli, M.; Mavromatis, A.; Chatzopoulou, P.; Karioti, A. Phenolic Profile by HPLC-PDA-MS of Greek Chamomile Populations and Commercial Varieties and Their Antioxidant Activity. *Foods* **2021**, *10*, 2345. https://doi.org/10.3390/foods10102345.

50. Dedić, S.; Džaferović, A.; Jukić, H. Chemical Composition and Antioxidant Activity of Water-Ethanol Extracts of Dandelion (*Taraxacum Officinale*). *Food Heal. Dis. Sci. J. Nutr. Diet.* **2022,** *11*, 8–14. https://hrcak.srce.hr/281051.

51. Biel, W.; Jaroszewska, A.; Łysoń, E.; Telesiński, A. The Chemical Composition and Antioxidant Properties of Common Dandelion Leaves Compared with Sea Buckthorn. *Can. J. Plant Sci.* **2017**, *97*, 1165–1174. https://doi.org/10.1139/cjps-2016-0409.

52. Palamutoglu, R.; Yalçın, S.; Kasnak, C. Comparison of Antioxidant Properties of Leaves of Plants Grown in Turkey. *World Sci. News* **2020**, *145*, 198–209. EISSN 2392-2192.

53. Petkova, N.; Ivanova, L.; Filova, G.; Ivanov, I.; Denev, P. Antioxidants and Carbohydrate Content in Infusions and Microwave Extracts from Eight Medicinal Plants. *J. Appl. Pharm. Sci.* **2017**, *7*, 55–61. https://doi.org/10.7324/JAPS.2017.71008.

54. Maaloul, S.; Ghzaiel, I.; Mahmoudi, M.; Mighri, H.; Pires, V.; Vejux, A.; Martine, L.; de Barros, J.P.P.; Prost-Camus, E.; Boughalleb, F.; et al. Characterization of *Silybum Marianum* and *Silybum Eburneum* Seed Oils: Phytochemical Profiles and Antioxidant Properties Supporting Important Nutritional Interests. *PLoS One* **2024**, *19*, 1–24. https://doi.org/10.1371/journal.pone.0304021.

55. Ivanov, V.; Pavlova, M. Comparative Study of the Antioxidant Properties of Silymarin and Milk Thistle Extracts. *Trakia J. Sci.* **2021**, *19*, 1–6. https://doi.org/10.15547/tjs.2021.01.001.

56. İnceören, N.; Emen, S.; Çeken Toptancı, B.; Kızıl, G.; Kızıl, M. In Vitro Inhibition of Advanced Glycation End Product Formation by Ethanol Extract of Milk Thistle (*Silybum Marianum* L.) Seed. *South Afr. J. Bot.* **2022**, *149*, 682–692. https://doi.org/10.1016/j.sajb.2022.06.062.

57. Kachel, M.; Krajewska, M.; Stryjecka, M.; Ślusarczyk, L.; Matwijczuk, A.; Rudy, S.; Domin, M. Comparative Analysis of Phytochemicals and Antioxidant Properties of Borage Oil (*Borago Officinalis* L.) and Milk Thistle (*Silybum Marianum* Gaertn). *Appl. Sci.* **2023**, *13*, 2560. https://doi.org/10.3390/app13042560.

59. El Mihyaoui, A.; Emilia, M.; Castillo, C.; Cano, A. Comparative Study of Wild Chamomile Plants from the North-West of Morocco: Bioactive Components and Total Antioxidant Activity. *J. Med. Plants Res.* **2021**, *5*, 431–441. https://doi.org/10.5897/JMPR2021.7159.

60. Helal, M.H.; Badr, S.E.; AbedElaaty, S.A. Chemical Characterization, Antioxidant, Anticancer and Hypolipidimic Activities of Chamomile (*Matricaria Chamomilla* L.). *Nutr. Res. Food Sci. J.* **2021**, *4*, 1–8. https://doi.org/10.31038/nrfsj.2021421.

61. Augspole, I.; Duma, M.; Ozola, B. Bioactive Compounds in Herbal Infusions. *Agron. Res.* **2018**, *16*, 1322–1330. https://doi.org/10.15159/AR.18.013.

Disclaimer/Publisher's Note: The statements, opinions and data contained in all publications are solely those of the individual author(s) and contributor(s) and not of MDPI and/or the editor(s). MDPI and/or the editor(s) disclaim responsibility for any injury to people or property resulting from any ideas, methods, instructions or products referred to in the content.
